# Objectively measured daily steps as an outcome in a clinical trial of chronic kidney disease: a systematic review

**DOI:** 10.1186/s12882-023-03412-x

**Published:** 2024-01-03

**Authors:** Liuyan Huang, Hui Wang, Yan Bai, Huachun Zhang, Fan Zhang, Yifei Zhong

**Affiliations:** 1 First Branch of Nephrology Department, Longhua Hospital Shanghai University of Traditional Chinese Medicine, No.725, Wanping South Road, Xuhui District, Shanghai, China; 2 Department of Anorectology, Longhua Hospital Shanghai University of Traditional Chinese Medicine, Shanghai, China; 3 Department of Nursing, Longhua Hospital Shanghai University of Traditional Chinese Medicine, Shanghai, China

**Keywords:** Chronic kidney disease, Daily steps, Physical activity, Pedometer, Systematic review

## Abstract

**Background:**

Physical inactivity is prevalent among individuals with chronic kidney disease (CKD) and is linked to unfavorable outcomes. In recent years, daily steps have emerged as a prominent target for interventions in clinical trials. The present study endeavors to scrutinize the effectiveness and/or efficacy of various interventions on daily steps in patients with full-spectrum CKD.

**Methods:**

In December 2022, a systematic search was conducted across three databases, namely PubMed, Embase, and Web of Science, and subsequently updated in June 2023. The inclusion criteria included randomized controlled studies, quasi-experimental studies, and single-arm trials that assessed an intervention’s impact on objectively measured daily steps in patients with chronic kidney disease. The Risk Of Bias In Non-randomized Studies-of Interventions (ROBINS-I) tool was used to assess the risk of bias in non-randomized controlled trials (RCT), while the Cochrane revised tool (ROB-2) was utilized for RCTs.

**Results:**

Seventeen studies were deemed eligible for inclusion in this review, with a focus on examining the efficacy and/or effectiveness of exercise training-based interventions (n = 10), daily step goal-oriented interventions (n = 4), mobile health (mHealth) interventions (n = 1), different dialysis modalities (n = 1), and a “Sit Less, Interact, Move More” intervention (n = 1). The studies exhibit variability in their characteristics and assessment tools, reflecting the findings’ heterogeneity. The results indicate that increasing physical activity levels remain challenging, as only a limited number of studies demonstrated significant improvements in participants’ daily step counts from baseline to endpoint.

**Conclusion:**

Clinical trials with daily steps as an outcome are still lacking in the CKD population. Well-designed clinical trials that objectively assess the physical activity of CKD patients are needed.

**Supplementary Information:**

The online version contains supplementary material available at 10.1186/s12882-023-03412-x.

## Introduction

Chronic kidney disease (CKD) is a significant public health concern [1]. This is due to the high societal costs associated with dialysis and kidney transplantation [2] and the elevated risk of cardiovascular events and mortality [3] among CKD patients. Consequently, many treatments and medications may be contraindicated or require dose adjustments [4].

Numerous studies have consistently demonstrated that individuals diagnosed with chronic kidney disease (CKD) exhibit lower physical activity levels than their healthy counterparts, leading to a chronically sedentary lifestyle linked to various negative health outcomes [5–7]. As a result, the United Kingdom Renal Association initiative advocates prioritizing promoting physical activity over exercise capacity assessment as it may significantly impact prognosis [8]. The daily step is a simple and direct measure of physical activity, which can be measured directly and inconspicuously in daily life and is a relevant patient-centered outcome [9].

Objective assessments of step counts offer distinct advantages over physical activity questionnaires, as they are not subject to recall bias, are more sensitive to change, and allow for collecting more granular data in real-world settings. This data can provide valuable insight into how individuals utilize their exercise capacity, commonly assessed through a 6-minute walk distance or cardiorespiratory exercise test [10, 11]. However, the validity and reliability of daily step count assessments are challenging, as they largely depend on the monitoring device selected and the standardization of the assessment [12].

Based on a systematic literature search, the current systematic review describes various interventions’ efficacy and/or effectiveness to enhance objectively measured endpoints that capture daily steps in patients with full-spectrum CKD.

## Methods

This system review is a subproject of the registered protocol (CRD42022385441) and written following the Preferred Reporting Items for Systematic Reviews and Meta-Analyses (PRISMA) reporting guidelines (Table S1). This study was exempt from ethics review because it is a pooled analysis of published data.

### Search strategy

PubMed, Embase and Web of Science were searched for articles published from inception to December 6, 2022, and updated in June 2023. An electronic database search was undertaken using combinations of Medical Subject Heading (MeSH) and keywords for “kidney disease,“ “renal failure,“ “physical activity,“ “daily step,“ and “sedentary behavior.“ The search strategy used is shown in Table S2.

### Eligibility criteria

This study aimed to identify English-language literature that reported the impact of interventions on objectively measured daily steps in patients with CKD. Eligibility criteria were based on Participant, Intervention, Comparator, Outcome, and Study design (PICOS) framework: (1) Participant: clinical trials that involved participants of any age with a diagnosis of CKD at any stage of treatment (pre-dialysis, peritoneal dialysis, hemodialysis, or kidney transplant recipients); studies that involved samples with multiple CKD stage were also included. (2) Intervention: interventions could include, but are not limited to, exercise training, physical activity counseling, educational programs, and self-management. (3) Outcome: daily steps assessed by an accelerometer, pedometer, or activity monitor. (4) Study Design: randomized controlled trials (RCTs), quasi-experimental studies., and single-arm studies were eligible.

### Study selection and data extraction

Two authors (HW and YB) screened the titles and abstracts based on the listed criteria. Indefinite articles were screened by reading the full text. Any discrepancies were discussed, and a consensus was reached to include or exclude a study.

The information on study design, sample size, patient characteristics, CKD stage, details of daily steps assessment, intervention, and daily steps outcomes (baseline and endpoints) were extracted from the article. Unless otherwise stated, mean ± standard deviation (SD) was used to report data (Table S3). Data described in the figures were obtained using Getdata software. If means and/or SDs were not available for included studies, the corresponding authors were contacted or calculated based on reported data (e.g., medians, quartiles) using recommended formulas [13, 14].

### Risk of bias assessment

Two authors (HW and YB) evaluated the included studies independently. The Risk of Bias in Non-randomized Studies-of Interventions (ROBINS-I) [15] tool was used to assess the risk of bias in non-RCTs, while the Cochrane revised tool (ROB-2) [16] was utilized for RCTs. Any inconsistencies were resolved through consensus with the review team.

## Results

### Study selection

**Fig. 1:**
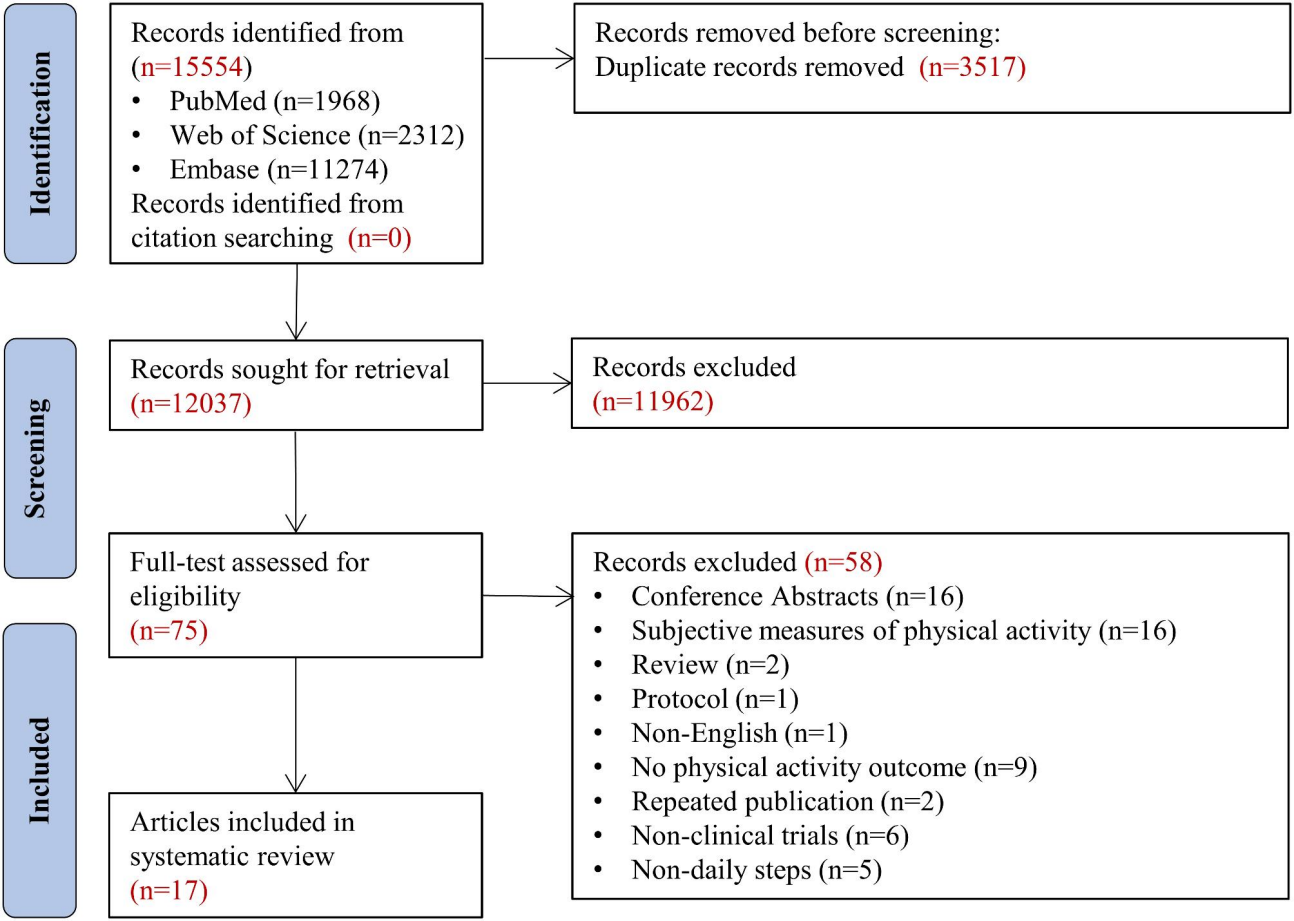
Study selection flow chart

We identified 15,554 articles through the search strategy and removed 3,517 duplicates. Seventy-five studies were identified for full-text selection through title and abstract screening. After excluding 58 studies that did not meet the eligibility criteria, 17 were deemed eligible and included in the analysis [17–33] (Fig. 1). See Table S4 for the list of excluded full-text articles and the corresponding reasons for exclusion.

### Study characteristics and quality assessment

**Fig. 2:**
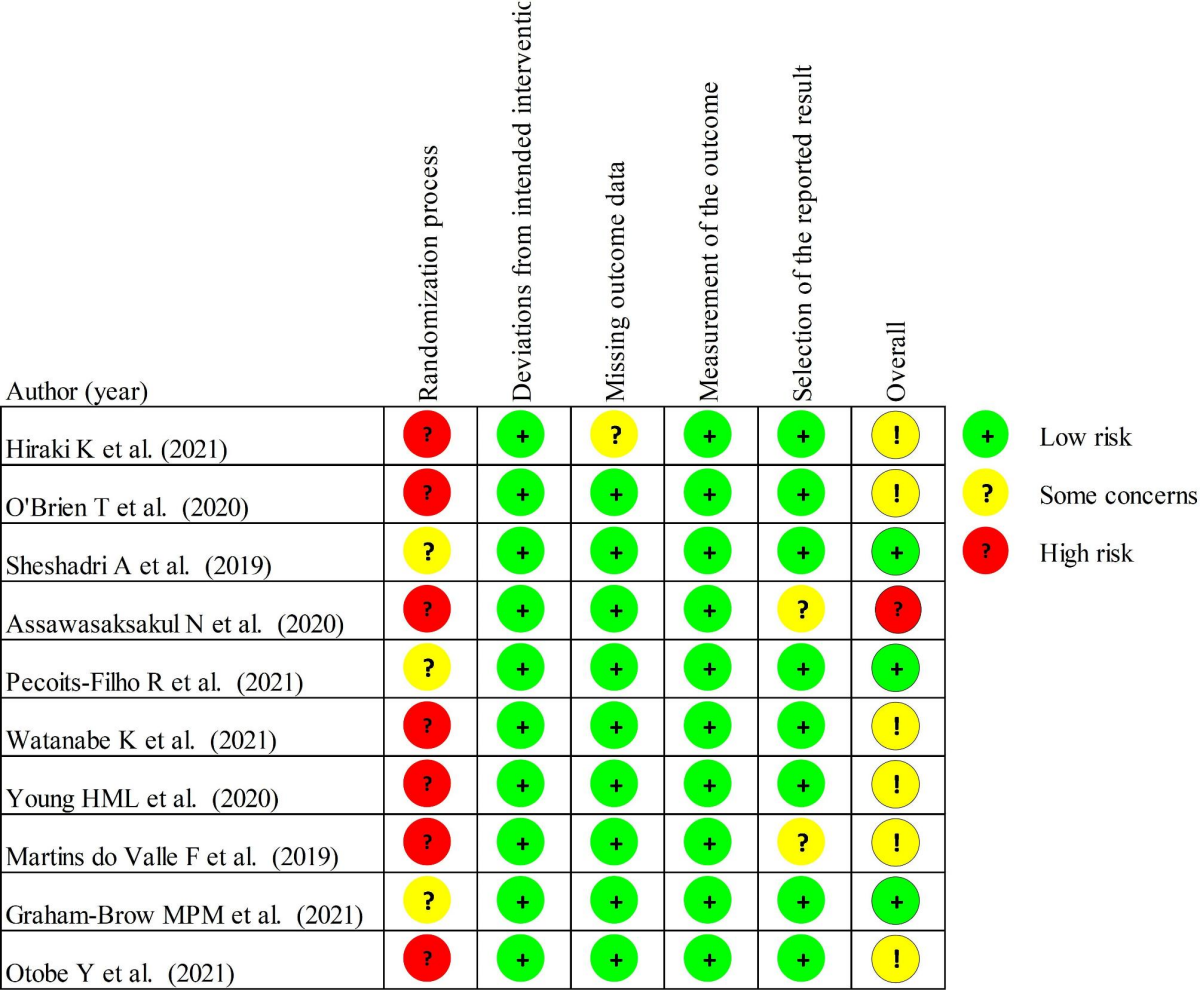
Risk assessment for inclusion in RCTs

Table 1 presents the characteristics of the studies included in this systematic review, encompassing a total sample size of 1032 participants, with each study having a range of 12 to 197 patients. Most studies recruited patients with hemodialysis-dependent CKD, while few included peritoneal dialysis patients. The age of the participants ranged from 46.9 to 78.4 years. Table 2; Fig. 2 summarize the risk of bias for the studies included in the review.

**Table 1:** The characteristic of included studies

First author [Ref.]	Year	Study design	Subjects (n)	Age (y)	Population	Intervention type	Duration	Physical activity measurement	Outcome
**Exercise training-based interventions**									
Masajtis-Zagajewska A et al. [19]	2019	Single-arm trial	39	46.9 ± 11.8	KTR (n = 24) and stage 3–4 CKD (n = 15)	Structured Physical Activity Program	12 weeks	3-axis SenseWear MF Armband accelerometer	Primary outcome
Assawasaksakul, N et al. [17]	2021	RCT	I: 6 C: 6	I:52.5 ± 12.9 C: 53.7 ± 17.2	HD	Intradialytic cycling exercise VS. usual care	24 weeks	Wrist-worn triaxial accelerometer	Primary outcome
Martins do Valle F et al. [18]	2020	RCT	I: 12 C: 12	I: 49.3 ± 12.4 C: 60.4 ± 10.6	HD	Intradialytic resistance exercise VS. usual care	12 weeks	Triaxial accelerometer	Primary outcome
Young HML et al. [24]	2020	RCT	I: 24 C: 27	I: 59 ± 13 C: 65 ± 11	HD	Intradialytic cycling exercise VS. usual care	24 weeks	SenseWear Armband Pro 3	Secondary outcome
Watanabe K et al. [23]	2021	RCT	I: 26 C: 27	I: 66.19 ± 13.05 C: 64.00 ± 12.95	PD	Intradialytic combination exercise VS. usual care	24 weeks	Three-axis accelerometer	Secondary outcome
Otobe Y et al. [30]	2021	RCT	I: 27 C: 26	I: 78.4 ± 6.4 C: 78.1 ± 7.4	Stage 3–4 CKD	Exercise training VS. usual care	24 weeks	Kenz Lifecorder Ex 1 axial accelerometer	Secondary outcome
Graham-Brown MPM et al. [26]	2021	RCT	I: 65 C: 65	I: 55.5 ± 15.5 C: 58.9 ± 14.9	HD	Intradialytic cycling exercise VS. usual care	24 weeks	Triaxial accelerometers	Secondary outcome
Hiraki K et al. [27]	2017	RCT	I: 14 C: 14	I: 69.0 ± 6.8 C: 67.8 ± 6.9	Stage 3–4 CKD	Home-based exercise program VS. usual care	84 weeks	Pedometers	Secondary outcome
Kontos P et al. [28]	2020	Quasi-experiment study	I: 10 C: 9	I: 72.7 ± 7.9 C: 73.9 ± 5.4	HD	Viewed Fit for Dialysis before participating in a 16-week exercise VS. usual care	16 weeks	Pedometers	Primary outcome
Bulckaen M et al. [25]	2011	Quasi-experiment study	18	N.P.	HD	Advised walking VS. supervised walking	6 months	Pedometers	Primary outcome
**Daily step goal-oriented interventions**									
Sheshadri A et al. [22]	2020	RCT	I: 30 C: 30	I: 56 [51–65] C: 60 [53–66] ^†^	Dialysis	Pedometers with weekly step goals VS. usual care	6 months	Pedometers	Primary outcome
Nowicki M et al. [29]	2010	Single-arm trial	33	58.3 ± 10.1	HD	Pedometers with weekly step goals	16 weeks	Pedometers	Primary outcome
O’Brien T et al. [20]	2020	RCT	I: 72 C: 26	I: 65.7 ± 4.9 C: 65.1 ± 4.0	KTR	SystemCHANGETM (Change Habits by Applying New Goals and Experience) + Activity Tracker intervention VS. attention	6 months	Number of daily steps recorded by Fitbit Charge 2	Secondary outcome
Malhotra R et al. (2023) [33]	2023	RCT	I: 28 C: 27	I: 62 ± 13 C: 61 ± 14	HD	Fitbit + feedback VS. Fitbit	12 weeks	Activity tracker	Primary outcome
**Mobile health (mHealth) interventions**									
Anand S et al. (2021) [31]	2021	RCT	I: 28 C: 28	I: 56.2 ± 12.3 C: 58.1 ± 9.9	Stage 3b-4 CKD	mHealth + exercise training VS. exercise training	16 weeks	Activity tracker	Secondary outcome
**Different dialysis modalities**									
Pecoits-Filho R et al. [21]	2021	RCT	HDF: 97 HD: 98	HDF: 52.6 ± 15.9 HD: 53.3 ± 14.3	HD	Hemodiafiltration VS. high-flux HD	6 months	Triaxial accelerometer	Primary outcome
**Sit Less, Interact, Move More intervention**									
Lyden K et al. [32]	2021	RCT	I: 54 C: 52	I: 69 ± 14 C: 69 ± 12	Stage 2–5 CKD	Sit Less, Interact, Move More VS. standard care	24 weeks	activPAL accelerometer	Primary outcome

Abbreviations: I, intervention; C, control; AE, aerobic exercise; RT, resistance training; CE, combined exercise; CKD, chronic kidney disease; HD, hemodialysis; KTR, kidney transplant recipient; HDF, hemodiafiltration; RCT, randomized control trial; N.P, not report

^†^ Median [quartiles]

**Table 2:** Risk assessment for inclusion in non-RCTs

Study	ROBINS-I (Non-Randomized Studies)							
	Confounding	Selection bias	Bias in measurement classification of intervention	Bias due to deviations intended interventions	Bias due to missing outcome data	Bias in measurement of outcomes	Bias in selection of reported results	Overall bias
Masajtis-Zagajewska A et al. (2019)	PY	PY	N	N	N	N	N	Moderate
Kontos P et al. (2021)	PY	N	N	N	Y	N	N	High
Nowicki M et al. (2010)	PY	PY	N	N	N	N	N	Moderate
Bulckaen M et al. (2011)	PY	PY	N	N	N	N	N	Moderate

**Note**: The response options regarding biases included Yes (Y), Probably Yes (PY), Probably No (PN), No (N) and No Information (NI). “Y” indicated a low risk of bias, “PY” indicated a moderate risk of bias, “PN” indicated a serious risk of bias, “N” indicated a critical risk of bias and “NI” indicated that was no information related to bias

**Fig. 3:**
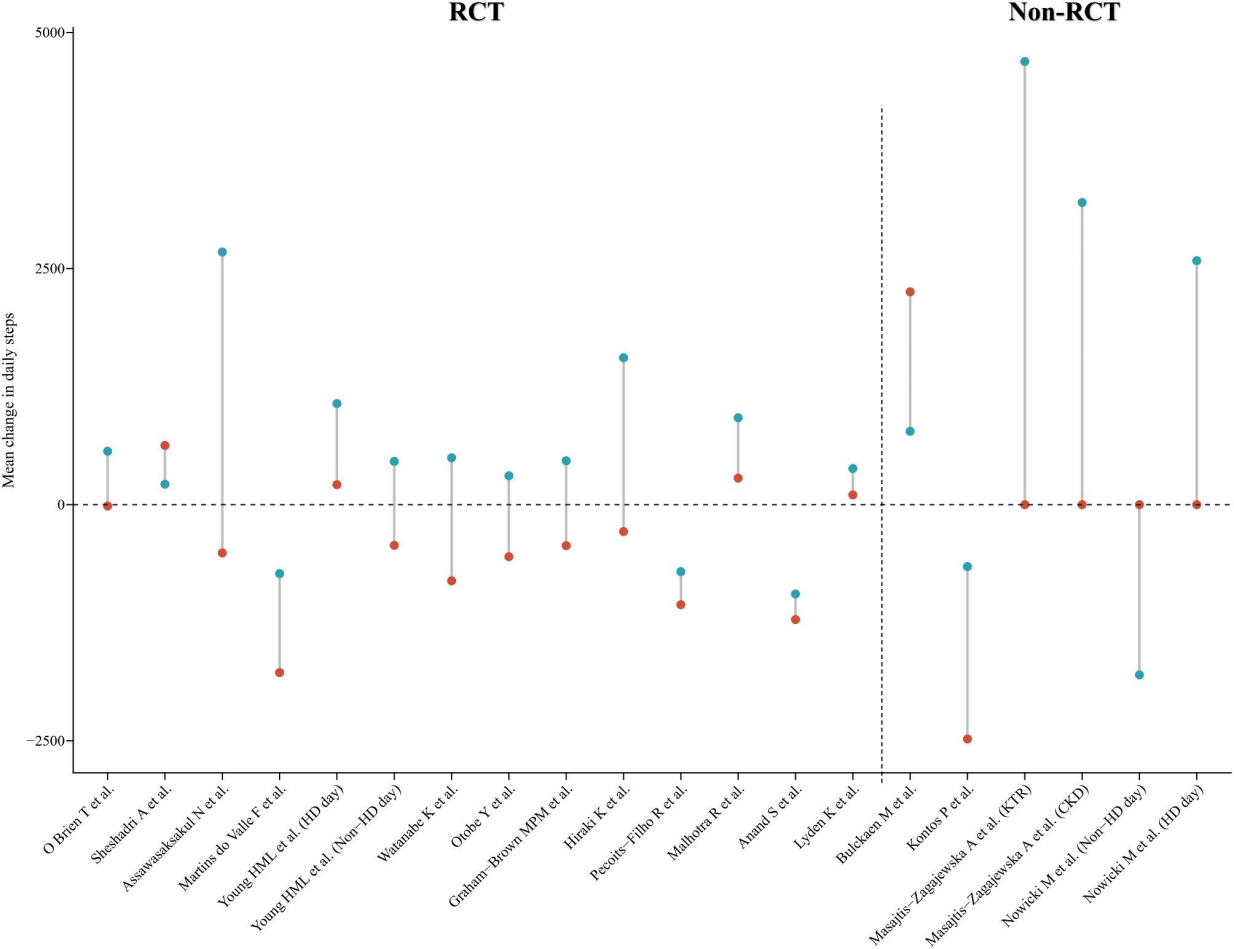
Daily steps pre-to-post intervention (red and blue circles indicate control and intervention, respectively)

According to the type of intervention described, we categorized the included studies into exercise training-based interventions (n = 10), pedometer-based walking programs (n = 4), mobile health (mHealth) interventions (n = 1), different dialysis modalities (n = 1), and Sit Less, Interact, Move More intervention (n = 1) (Table 1). Figure 3 depicts the mean difference in endpoint-baseline daily step counts for the included studies.

### Exercise training-based interventions

Seven RCTs, two quasi-experiment studies, and one single-arm trial explored the role of exercise-based interventions in modifying daily steps in patients with CKD. Six of these studies used an accelerometer, three used Pedometers, and one used SenseWear Armband.

#### Randomized controlled trials

Three RCTs were conducted to assess the impact of combined exercise compared to usual care. In one of these studies, conducted by Watanabe K et al. [23], 71 patients with CKD who underwent peritoneal dialysis were randomly assigned to unsupervised, home-based combined exercise or usual care groups. The exercise regimen included walking, resistance exercises, and stretching, while the control group received only stretching training. A three-axis accelerometer was used for monitoring physical activity. The daily step counts increased from 4820.5 ± 2698.6 to 5710.7 ± 2698.8 steps/day in 26 peritoneal dialysis patients and then decreased to 5316.8 ± 2254.5 steps/day in the sixth month while gradually decreasing in the control group (Figure S1a). Another home-based combined exercise for patients with CKD stages 3–4 reported a significant increase in daily steps (6725.3 ± 3152.4 steps/day to 8281.4 ± 3108.8 steps/day; *P* = 0.04) [27] (Figure S1b). Otobe Y et al. [30] recruited elderly patients with CKD stages 3–4, and 23 participants completed group exercise training supervised by a physiotherapist at a rehabilitation center and increased their daily step count from 3540 (2061, 6089) steps/day to 3757 (2324, 6477) steps/day after a 24-week intervention, with a similar decrease in the control group (Figure S1c).

Three RCTs added an aerobic exercise program to usual care. Graham-Brown MPM and colleagues [26] evaluated the effects of a 6-month progressive cycling exercise program on dialysis in 130 hemodialysis patients. Tri-axial accelerometers were used to measure physical activity over seven consecutive days. Data obtained from 66 participants showed that compared to baseline, patients in the exercise group increased their daily step count from 2710 ± 1945 steps/day to 3175 ± 2610 steps/day, while the control group decreased from 3514 ± 4099 steps/day to 3080 ± 3445 steps/day, and the difference between the two groups was also not statistically significant (732 steps/day; 95% CI -75 to 1539) (**Figure S2a**). Similar results were reported by Young HML et al. [24]: the overall difference between the progressive cycling and control groups was 859 steps/day (95% CI -825 to 2543) on hemodialysis days and 888 steps/day (95% CI -84 to 1861) on non-hemodialysis days (**Figure S2b**). The results of another small sample (n = 12) study [17] showed a significant increase in daily steps from 5613 (2949.8, 8175.7) to 8725.1 (6299.7, 9908.7) (*P* = 0.046) over six months in the exercise group, while the control group decreased from 5398.9 (3232.6, 7001.9) to 4252.1 (2745.0, 6856.1) (*P* = 0.046) (**Figure S2c**).

Martins do Valle F and colleagues [18] evaluated the effects of an intradialytic resistance training program in 24 hemodialysis patients. Daily steps were measured using a triaxial accelerometer for seven consecutive days (including three dialysis days and four non-dialysis days). Resistance training (12 weeks, three times/week) was based on both lower limbs and in the contralateral arteriovenous fistula upper limb. After 12 weeks, participants showed a significant improvement in 6-minute walking distance. However, the two groups had no statistically significant difference in the pre-and post-intervention change in daily step count.

#### Nonrandomized studies

A single-arm study [19] found that after a 12-week physiotherapist-supervised, free-choice aerobic exercise (Nordic walking, jogging, cycling, or swimming) program, daily steps and total energy expenditure increased significantly in 24 kidney transplant recipients and 15 patients with stage 3–4 CKD (**Figure S3a**).

A study by Bulckaen et al. [25] evaluated the effects of a 6-month center-based aerobic exercise and home-based walking program on physical activity in 13 hemodialysis patients. Compared to baseline, participants in the home-based group showed a significant increase in daily steps, as indicated by pedometers after months 3 and 6, whereas there was no substantial change in the center-based group (**Figure S3b**).

Kontos and colleagues [28] designed a quasi-experiment that introduced the Fit for Dialysis film, based on which an exercise program was developed for ten people > 65 years of age with hemodialysis, with 20–30 min of aerobic exercise 2–3 times per week (40–90 min per week) and 10–15 min of resistance training 2–3 times per week (20–45 min per week), with two physiotherapy assistant were responsible for supervision. However, at the end of the 28-week intervention, no significant differences in the participants’ daily step counts were detected (**Figure S3c**).

### Daily step goal-oriented interventions

Three RCTs and one single-arm trial evaluated the effect of daily step goal-oriented interventions on physical activity.

#### Randomized controlled trials

Sheshadri and colleagues [22] incorporated goal-setting theory and assessed physical activity by pedometer with a goal of a “10% increase in steps compared to the previous week”. Compared to baseline, 27 patients with CKD stages 3–4 increased their daily step count by 2132 ± 2771 steps/day at month three and then decreased by 1596 ± 2369 steps/day at months 3–6 (**Figure S4a**). Surprisingly, remotely guided interventions, including lifestyle behavior change via pedometer feedback or individualized lifestyle change strategies, did not result in significant changes in either moderate daily physical activity or daily steps after six months compared to baseline. O’Brien et al. reported [20] that participants’ daily steps in the first three months gradually increased and then slowly decreased, but the difference between the two groups was not statistically significant (**Figure S4b**). Malhotra et al. [33] recruited 56 patients with hemodialysis-dependent CKD; all participants wore a Fitbit Charge 2 tracker, and one group received additional face-to-face goal-setting counseling. Personalized goal setting focused on increasing daily and weekly step goals (typically a 10% increase in steps from baseline). During the 12-week intervention period, participants in the intervention group had a significant increase in daily steps from baseline to week 12 compared with the comparison group (920 ± 580 vs. 281 ± 186; between-group difference Δ 639 ± 538 steps; *P* < 0.05). The magnitude of change in step count differences was most significant in the first 4 weeks (Δ1126 ± 517 VS. Δ494 ± 281 steps) and decreased slightly thereafter but maintained statistically significant differences between groups throughout the study period (**Figure S4c**).

#### Nonrandomized studies

Nowicki et al. [29] included 33 hemodialysis patients with physical activity levels recorded by pedometers seven times over four months. Compared to baseline, daily steps between 2-midweek dialysis sessions and spontaneous step counts on non-dialysis days were significantly increased (Figure S5).

### Mobile health (mHealth) interventions

One RCT examined the effectiveness of mobile phone-based (mHealth) interventions to change physical activity in patients with CKD. This studies used the wearable Garmin Vivofit 3 activity tracker to quantify physical activity.

#### Randomized controlled trials

Anand et al. [31] conducted a comparative study to evaluate the impact of mHealth combined twice weekly directed exercise session versus only exercise sessions on daily step counts in 56 patients with CKD stage 3b-4. The intervention lasted eight weeks, followed by an 8-week passive follow-up period. The results indicated that the mean step count decreased in both groups and exhibited no significant differences over time (Figure S6).

#### Nonrandomized studies

There were no nonrandomized studies available for review.

### Different dialysis modalities

A study compared the effects of hemodiafiltration or high-flux hemodialysis treatment on physical activity in hemodialysis-dependent CKD patients.

#### Randomized controlled trials

Pecoits-Filho et al. [21] conducted a study involving 197 hemodialysis patients, wherein physical activity was measured using a waist-mounted triaxial accelerometer seven days before each study visit (baseline, three months, and six months). Results indicated that at the 3-month mark, participants in the hemodiafiltration group exhibited a slight increase in step counts, whereas those in the high-flux hemodialysis group experienced a decrease in steps/24 h (hemodiafiltration 5303 ± 3442 VS. high-flux hemodialysis 4249 ± 2734, *P* = 0.03). However, these differences were not sustained at the 6-month follow-up (Figure S7).

#### Nonrandomized studies

There were no non-randomized studies available for review.

### Sit less, interact, move more intervention

One study designed an individualized program for sedentary and step durations.

#### Randomized controlled trials

Lyden et al. [32] conducted a study involving 106 patients diagnosed with stages 2–5 CKD. The intervention group received feedback on their sedentary behavior through an accelerometer display and were instructed to engage in low-intensity activities by standing up from a sitting/lying awake position at least once per hour. The control group received standard care, which included encouragement to complete 150 min of moderate/vigorous activity per week. The intervention group experienced a significant increase in daily steps at week 20 (mean difference: 1265; 95% CI 518 to 2012), which decreased by week 24.

#### Nonrandomized studies

There were no non-randomized studies available for review.

## Discussion

### Summary of main results

In recent years, nephrologists have progressively highlighted and advocated for clinical investigations involving physical activity, specifically daily step count, as a measurable outcome [34]. This literature review identifies 17 interventional trials concentrating on exercise training-based interventions, daily step goal-oriented interventions, mobile health (mHealth) interventions, various dialysis modalities, and a “Sit Less, Interact, Move More” intervention reporting objectively measured daily steps outcomes. However, the heterogeneity of these studies presents challenges in drawing generalizable conclusions.

In the included studies, no specific devices were used to objectively assess daily step counts, although the most used tools currently include only accelerometers, pedometers, or activity monitors. It is crucial, however, that researchers are aware of the accuracy and reliability of the equipment used. The selection of measurement devices should be based on these characteristics, considering cost, user acceptance, and study design (e.g., physical activity as an outcome measure versus physical activity monitoring as part of an intervention).

### Comparison of interventions oriented to change in step counts

In 50% of the studies assessing intervention strategies centered on exercise training, the primary outcome was the number of daily steps. Patients with CKD commonly exhibit low cardiorespiratory fitness, which frequently contributes to physical inactivity [35]. While not specifically designed to alter physical activity levels, exercise training interventions primarily seek to enhance an individual’s exercise tolerance, with concomitant improvements in physiological function potentially leading to behavioral changes. Several studies have reported significant alterations in the number of steps participants took [17, 27], indicating a potential impact of interventions on physical activity levels. However, whether these interventions can effectively promote the maintenance of physical activity remains a subject of ongoing research, with important implications for clinical practice. This is due to the critical role of adherence in establishing a connection between exercise training and sustained physical activity [36].

A qualitative study reported that most CKD patients believe that appropriate physical activity makes them feel better and positively impacts their health [37]. In another study, the top priority for older CKD patients was maintaining independence through physical activity, whereas for younger patients, longevity and transplant candidacy were the most important motivating factors [38]. Evidence from cohort studies suggests that adherence to physical activity guidelines is significantly associated with all-cause mortality (HR: 0.49; 95% CI: 0.38–0.63), malignancy mortality (HR: 0.30; 95% CI: 0.17–0.52), and albumin-to-creatinine ratio (OR: -0.27; 95% CI: -0.39 to -0.15) [39]. A retrospective study showed that ‘non-completers’ (successful completion of a pragmatic renal rehabilitation program more than 50%) of renal rehabilitation had a 1.6-fold (95% CI 1.00-2.58) greater risk of a combined event [40]. Thus, improving patient compliance is essential to ensure that physical activity is delivered.

Research on daily step goal-oriented interventions has demonstrated a higher likelihood of observing an increase in daily steps. However, variability exists between outcomes. Notably, pedometer-based interventions are often provided as stand-alone measures, and thus, the existing body of literature fails to furnish definitive proof that increased daily step counts elicit supplementary alterations in physical activity among individuals afflicted with CKD. Indeed, a high-quality RCT designed by Sheshadri and colleagues found that the increase in daily steps was maintained for only three months and then returned to the starting point [22]; as the authors state, Walking does not capture the full spectrum of physical activity.

The interventions incorporated into telemedicine primarily focused on altering participants’ daily lives rather than explicitly targeting behavioral modifications aimed at increasing physical activity. As a result, no significant changes in physical activity were observed as secondary outcomes. This implies that alterations in daily activities did not translate into increased physical activity. Nevertheless, this finding remains a subject of further exploration, considering the growing popularity and rapid evolution of telemedicine [41].

In light of the potential influence of dialysis modality and/or dose on physical activity among hemodialysis-dependent CKD patients, a multicenter RCT carried out by Pecoits-Filho et al. [21] revealed that high-volume online-hemodiafiltration did not result in a statistically significant alteration in daily step count over a period of six months. However, it is noteworthy that individuals in the hemodiafiltration group exhibited an increase in daily steps compared to their baseline in the third month and were significantly higher than those in the hemodialysis group. Nonetheless, researchers believe that the program’s systematic and standardized collection of accelerated measurement data helps to understand the granular level of physical activity in dialysis patients concerning demographics, clinical characteristics, and treatment plans and provides a basis for planning physical activity interventions for dialysis patients.

CKD patients tend to engage in sedentary behaviors and exhibit physical inactivity [42, 43], strongly associated with a higher risk of all-cause mortality [6, 44]. Lyden and colleagues [32] conducted an RCT to address this issue and introduce a novel approach to mitigate the negative impact of a sedentary lifestyle in CKD patients. The intervention encouraged participants to stand up from a sedentary position at least once per hour and engage in light physical activity. According to Kim et al. [45], the present study demonstrates that reducing sedentary behavior leads to clinically significant enhancements in walking hours among patients with CKD, highlighting the need to combine sustained patient engagement with ongoing feedback and reinforcement.

In addition to the above, a cross-sectional study based on National Health and Nutrition Examination Survey noted that participants with the longest sedentary time had a 1.69-fold (95% CI: 1.27–2.24) higher risk of depression than CKD patients with shorter sedentary time [46]. Results from another large-sample cohort study showed that replacing 1 h/day of sedentary time with an equivalent amount of vigorous-intensity physical activity was associated with a 25% lower risk of depression in those with longer sedentary time (> 6 h/day) (HR: 0.85; 95% CI 0.75–0.97) [47]. An observational study by Zhu et al. reported significantly lower odds of a major depressive episode in patients with CKD who were consistently active than those who were consistently inactive (OR: 0.102, 95% CI 0.022–0.467) [48]. It is worth noting that recent guidelines state that CKD patients should engage in physical activity, and some physical activity is better than none. The benefits of increasing physical activity in CKD patients are, and are not limited to, improvements in mental health, including depression and anxiety symptoms [49].

### Implications

Regarding study characteristics, most of the literature, in terms of current included studies, has been directed at hemodialysis patients. In contrast, relatively few studies have been conducted in pre-dialysis and kidney transplant recipients [50], and trials recruiting peritoneal dialysis patients are even more lacking [51]. The reasons for this are twofold: (1) hemodialysis patients spend a few hours on dialysis three days a week and usually have little to do during their treatment, so intradialytic exercise, interventions included in most trials reported, is considered relatively convenient and time-saving. (2) peritoneal dialysis usually needs to be performed at home and is relatively frequent, possibly several times daily. This may limit patients’ time and energy to engage in exercise. Studies based on exercise interventions have reported benefits for patients with end-stage renal disease treated with hemodialysis [52]; however, there are few studies explicitly involving patients on peritoneal dialysis, and there is a lack of well-designed randomized controlled trials that would allow for significant and valid evidence-based research on exercise and peritoneal dialysis patient-oriented outcome measures [51]. The first exercise guideline focusing on peritoneal dialysis patients, published in 2019 by the International Society for Peritoneal Dialysis in conjunction with the Global Renal Exercise Network, also does not outline evidence-based advice for changing physical activity in peritoneal dialysis patients [53].

Implementing interventions that maximize the benefits of physical activity in patients with CKD remains a daunting challenge, but there is reason for optimism. In the included trials, most participants benefited from short-term interventions. Thus, the discrepancy in the results of many studies may indicate the challenge of conducting large, long-term clinical trials in CKD patients rather than evidence of ineffective interventions. Despite increasing use of physical activity endpoints in CKD trials, there is no consensus on the best measure. Indeed, endpoints are inconsistent across CKD trials, making translation into clinical practice difficult.

Based on the above analysis, several recommendations can be made for future research. The first recommendation is that adapting previously validated strategies associated with hemodialysis trials to a representative sample of the peritoneal dialysis population may be a possible future course of action. After all, there is currently little knowledge surrounding exercise in peritoneal dialysis patients. A recent international-wide survey showed that most clinical professionals agree that organized exercise programs benefit patients undergoing peritoneal dialysis treatment and that more exercise could be performed [54]. Second, a concern with wearable devices is whether individuals with CKD will adhere to them long-term. It has been reported that usage of wearable devices declines over time, with approximately 25–50% of individuals discontinuing use within six months [55]. This may suggest that device is a first step in helping individuals develop an awareness of physical activity, but other techniques (e.g., cognitive-behavioral interventions) may need to be tailored to promote sustained use over a longer period. Alternatively, researchers could utilize a window of opportunity during the intervention to integrate the device into daily life, and a deeper understanding of the wearable device, such as wearing time and accessing and interpreting data, may help with patient insight, motivation, and engagement [56].

### Limitation

There are several limitations in this review. Firstly, daily steps may be a secondary outcome in some studies; however, finding these studies was difficult, as the titles and abstracts do not necessarily describe physical activity assessment in the articles. Despite a rigorous screening process, we cannot completely exclude the possibility that a few studies were not identified. Secondly, the scope of our review was limited to literature published exclusively in English, which may have resulted in the exclusion of significant studies published in other languages. Thirdly, our review solely focused on clinical trials, and only a select number of noteworthy findings were reported.

## Conclusion

Although the tools used to objectively assess daily steps in patients with CKD are mostly pedometers and accelerometers, the current clinical intervention trials still show heterogeneous results. In particular, it should be noted that the gap between research on improving physical activity in CKD and similar literature in chronic obstructive pulmonary disease [57] and cardiovascular disease [58] is still huge, further well-designed trials are needed.

### Electronic supplementary material

Below is the link to the electronic supplementary material.

**Supplementary Material 1: Table S1** The PRISMA 2020 Checklist. **Table S2** Search detailed for databases. **Table S3** Physical activity outcomes extracted from included studies. **Table S4** Reasons for excluding studies from the final analysis after full text assessment. **Figure S1** Results from three RCTs based on combined exercise training interventions. **Figure S2** Results from three RCTs based on aerobic exercise training interventions. **Figure S3** Results from three non-RCTs based on exercise training interventions. **Figure S4** Results of three RCTs from a daily step goal-oriented interventions. **Figure S5** Results of a single-arm trial from a daily step goal-oriented intervention. **Figure S6** Results of an RCT from the mHealth combined exercise. **Figure S7** Results from an RCT comparing different dialysis modalities

## Data Availability

The data covered in the manuscript are provided in the text or supplementary material.

## Declarations

### Data sharing statement

This systematic review was a secondary analysis of raw data from published original articles. All the data used for the study are included in the manuscript and supplementary material.

## References

[1] Romagnani P, Remuzzi G, Glassock R, Levin A, Jager KJ, Tonelli M, Massy Z, Wanner C, Anders HJ (2017). Chronic Kidney Disease. Nat Rev Dis Primers.

[2] Chen TK, Knicely DH, Grams ME (2019). Chronic Kidney Disease diagnosis and management: a review. JAMA.

[3] Webster AC, Nagler EV, Morton RL, Masson P (2017). Chronic Kidney Disease. Lancet.

[4] de Boer IH, Khunti K, Sadusky T, Tuttle KR, Neumiller JJ, Rhee CM, Rosas SE, Rossing P, Bakris G (2022). Diabetes management in chronic Kidney Disease: a consensus report by the American Diabetes Association (ADA) and Kidney Disease: improving global outcomes (KDIGO). Kidney Int.

[5] Bruinius JW, Hannan M, Chen J, Brown J, Kansal M, Meza N, Saunders MR, He J, Ricardo AC, Lash JP (2022). Self-reported physical activity and Cardiovascular events in adults with CKD: findings from the CRIC (chronic renal insufficiency cohort) study. Am J Kidney Dis.

[6] Kuo CP, Tsai MT, Lee KH, Lin YP, Huang SS, Huang CC, Tseng WC, Tarng DC (2022). Dose-response effects of physical activity on all-cause mortality and major cardiorenal outcomes in chronic Kidney Disease. Eur J Prev Cardiol.

[7] Rampersad C, Brar R, Connelly K, Komenda P, Rigatto C, Prasad B, Bohm C, Tangri N (2021). Association of Physical Activity and Poor Health Outcomes in patients with Advanced CKD. Am J Kidney Dis.

[8] Clinical Practice Guideline: Exercise and Lifestyle in Chronic Kidney Disease [https://ukkidney.org/sites/renal.org/files/Exercise and Lifestyle in CKD clinical practice guideline33_v4_FINAL_0.pdf] 10.1186/s12882-021-02618-1 PMC8862368 35193515

[9] Zelle DM, Klaassen G, van Adrichem E, Bakker SJ, Corpeleijn E, Navis G (2017). Physical Inactivity: a risk factor and target for intervention in renal care. Nat Rev Nephrol.

[10] Atkin AJ, Gorely T, Clemes SA, Yates T, Edwardson C, Brage S, Salmon J, Marshall SJ, Biddle SJ (2012). Methods of measurement in epidemiology: sedentary Behaviour. Int J Epidemiol.

[11] Bassett DR, Wyatt HR, Thompson H, Peters JC, Hill JO (2010). Pedometer-measured physical activity and health behaviors in U.S. adults. Med Sci Sports Exerc.

[12] Kelly P, Fitzsimons C, Baker G (2016). Should we reframe how we think about physical activity and sedentary behaviour measurement? Validity and reliability reconsidered. Int J Behav Nutr Phys Act.

[13] Luo D, Wan X, Liu J, Tong T (2018). Optimally estimating the sample mean from the sample size, median, mid-range, and/or mid-quartile range. Stat Methods Med Res.

[14] Wan X, Wang W, Liu J, Tong T (2014). Estimating the sample mean and standard deviation from the sample size, median, range and/or interquartile range. BMC Med Res Methodol.

[15] Sterne JA, Hernan MA, Reeves BC, Savovic J, Berkman ND, Viswanathan M, Henry D, Altman DG, Ansari MT, Boutron I (2016). ROBINS-I: a tool for assessing risk of bias in non-randomised studies of interventions. BMJ.

[16] Sterne JAC, Savović J, Page MJ, Elbers RG, Blencowe NS, Boutron I, Cates CJ, Cheng HY, Corbett MS, Eldridge SM (2019). RoB 2: a revised tool for assessing risk of bias in randomised trials. BMJ.

[17] Assawasaksakul N, Sirichana W, Joosri W, Kulaputana O, Eksakulkla S, Ketanun C, Kittiskulnam P, Chantadisai M, Takkavatakarn K, Susantitaphong P (2021). Effects of intradialytic cycling exercise on daily physical activity, physical fitness, body composition, and clinical parameters in high-volume online hemodiafiltration patients: a pilot randomized-controlled trial. Int Urol Nephrol.

[18] Martins do Valle F, Valle Pinheiro B, Almeida Barros AA, Ferreira Mendonça W, de Oliveira AC, de Oliveira Werneck G, de Paula RB, Moura Reboredo M (2020). Effects of intradialytic resistance training on physical activity in daily life, muscle strength, physical capacity and quality of life in hemodialysis patients: a randomized clinical trial. Disabil Rehabil.

[19] Masajtis-Zagajewska A, Muras K, Nowicki M (2019). Effects of a structured physical activity program on habitual physical activity and body composition in patients with chronic Kidney Disease and in kidney transplant recipients. Exp Clin Transplant.

[20] O’Brien T, Russell CL, Tan A, Mion L, Rose K, Focht B, Daloul R, Hathaway D (2020). A pilot randomized controlled trial using SystemCHANGE™ Approach to increase physical activity in older kidney transplant recipients. Prog Transpl.

[21] Pecoits-Filho R, Larkin J, Poli-de-Figueiredo CE, Cuvello-Neto AL, Barra ABL, Gonçalves PB, Sheth S, Guedes M, Han M, Calice-Silva V (2021). Effect of hemodiafiltration on measured physical activity: primary results of the HDFIT randomized controlled trial. Nephrol Dial Transplant.

[22] Sheshadri A, Kittiskulnam P, Lazar AA, Johansen KL (2020). A walking intervention to increase Weekly steps in Dialysis patients: a pilot randomized controlled trial. Am J Kidney Dis.

[23] Watanabe K, Kamijo Y, Yanagi M, Ishibashi Y, Harada T, Kohzuki M (2021). Home-based exercise and bone mineral density in peritoneal dialysis patients: a randomized pilot study. BMC Nephrol.

[24] Young HML, March DS, Highton PJ, Graham-Brown MPM, Churchward DC, Grantham C, Goodliffe S, Jones W, Cheung MM, Greenwood SA (2020). Exercise for people living with frailty and receiving haemodialysis: a mixed-methods randomised controlled feasibility study. BMJ Open.

[25] Bulckaen M, Capitanini A, Lange S, Caciula A, Giuntoli F, Cupisti A (2011). Implementation of exercise training programs in a hemodialysis unit: effects on physical performance. J Nephrol.

[26] Graham-Brown MPM, March DS, Young R, Highton PJ, Young HML, Churchward DR, Dungey M, Stensel DJ, Bishop NC, Brunskill NJ (2021). A randomized controlled trial to investigate the effects of intra-dialytic cycling on left ventricular mass. Kidney Int.

[27] Hiraki K, Shibagaki Y, Izawa KP, Hotta C, Wakamiya A, Sakurada T, Yasuda T, Kimura K (2017). Effects of home-based exercise on pre-dialysis chronic Kidney Disease patients: a randomized pilot and feasibility trial. BMC Nephrol.

[28] Kontos P, Colobong R, Grigorovich A, Palma Lazgare LI, Binns M, Alibhai S, Parsons T, Nesrallah G, Jassal SV, Thomas A (2021). Fit for Dialysis: a prospective 2-site parallel intervention trial of a filmed research-based drama to increase exercise amongst older hemodialysis patients. Int Urol Nephrol.

[29] Nowicki M, Murlikiewicz K, Jagodzińska M (2010). Pedometers as a means to increase spontaneous physical activity in chronic hemodialysis patients. J Nephrol.

[30] Otobe Y, Yamada M, Hiraki K, Onari S, Taki Y, Sumi H, Hachisuka R, Han W, Takahashi M, Suzuki M (2021). Physical Exercise improves cognitive function in older adults with stage 3–4 chronic Kidney Disease: a Randomized Controlled Trial. Am J Nephrol.

[31] Anand S, Ziolkowski SL, Bootwala A, Li J, Pham N, Cobb J, Lobelo F (2021). Group-based Exercise in CKD Stage 3b to 4: a Randomized Clinical Trial. Kidney Med.

[32] Lyden K, Boucher R, Wei G, Zhou N, Christensen J, Chertow GM, Greene T, Beddhu S (2021). Targeting sedentary behavior in CKD: a Pilot and Feasibility Randomized Controlled Trial. Clin J Am Soc Nephrol.

[33] Malhotra R, Rahimi S, Agarwal U, Katz R, Kumar U, Garimella PS, Gupta V, Chopra T, Kotanko P, Ikizler TA et al. The impact of a wearable activity tracker and structured feedback program on physical activity in hemodialysis patients: the Step4Life pilot randomized controlled trial. Am J Kidney Dis 2023. 10.1053/j.ajkd.2022.12.011 PMC10962931 36801430

[34] Wilkinson TJ, Smith AC (2021). Physical activity and living well with Kidney Disease. Nat Rev Nephrol.

[35] Roshanravan B, Gamboa J, Wilund K (2017). Exercise and CKD: skeletal muscle dysfunction and practical application of Exercise to prevent and treat physical impairments in CKD. Am J Kidney Dis.

[36] Clyne N, Anding-Rost K (2021). Exercise training in chronic kidney disease-effects, expectations and adherence. Clin Kidney J.

[37] Clarke AL, Young HM, Hull KL, Hudson N, Burton JO, Smith AC (2015). Motivations and barriers to exercise in chronic Kidney Disease: a qualitative study. Nephrol Dial Transplant.

[38] Moorman D, Suri R, Hiremath S, Jegatheswaran J, Kumar T, Bugeja A, Zimmerman D (2019). Benefits and barriers to and desired outcomes with Exercise in patients with ESKD. Clin J Am Soc Nephrol.

[39] Qu X, Tong X, Hou X, Zhang J, Hou L, Chen J (2022). Trends in Adherence to recommended physical activity and its Association with Mortality and Disease Progression among US adults with chronic Kidney Disease. Am J Nephrol.

[40] Greenwood SA, Castle E, Lindup H, Mayes J, Waite I, Grant D, Mangahis E, Crabb O, Shevket K, Macdougall IC (2019). Mortality and morbidity following exercise-based renal rehabilitation in patients with chronic Kidney Disease: the effect of programme completion and change in exercise capacity. Nephrol Dial Transplant.

[41] Wieringa FP, Broers NJH, Kooman JP, Van Der Sande FM, Van Hoof C (2017). Wearable sensors: can they benefit patients with chronic Kidney Disease?. Expert Rev Med Devices.

[42] Wilkinson TJ, Clarke AL, Nixon DGD, Hull KL, Song Y, Burton JO, Yates T, Smith AC (2021). Prevalence and correlates of physical activity across Kidney Disease stages: an observational multicentre study. Nephrol Dial Transplant.

[43] Takahashi A, Hu SL, Bostom A (2018). Physical activity in kidney transplant recipients: a review. Am J Kidney Dis.

[44] Bruinius JW, Hannan M, Chen J, Brown J, Kansal M, Meza N, Saunders MR, He J, Ricardo AC, Lash JP (2022). Self-reported physical activity and Cardiovascular events in adults with CKD: findings from the CRIC (chronic renal insufficiency cohort) study. Am J Kidney Dis.

[45] Kim TY, Roshanravan B (2021). Moving beyond Sedentarism in CKD. Clin J Am Soc Nephrol.

[46] Liu L, Yan Y, Qiu J, Chen Q, Zhang Y, Liu Y, Zhong X, Liu Y, Tan R (2023). Association between sedentary behavior and depression in US adults with chronic Kidney Disease: NHANES 2007–2018. BMC Psychiatry.

[47] Cao Z, Xu C, Zhang P, Wang Y (2022). Associations of sedentary time and physical activity with adverse health conditions: outcome-wide analyses using isotemporal substitution model. EClinicalMedicine.

[48] Zhu FX, Zhang XY, Ding XK, Han B (2017). Protective effect of regular physical activity on major depressive episodes in patients with early stages of chronic Kidney Disease. Ren Fail.

[49] Baker LA, March DS, Wilkinson TJ, Billany RE, Bishop NC, Castle EM, Chilcot J, Davies MD, Graham-Brown MPM, Greenwood SA (2022). Clinical practice guideline exercise and lifestyle in chronic Kidney Disease. BMC Nephrol.

[50] Barcellos FC, Santos IS, Umpierre D, Bohlke M, Hallal PC (2015). Effects of exercise in the whole spectrum of chronic Kidney Disease: a systematic review. Clin Kidney J.

[51] Maia Neves Menezes JI, Lopes Pereira LA (2022). Physical exercise and peritoneal dialysis: an area yet to be explored. Nefrologia (Engl Ed).

[52] Bernier-Jean A, Beruni NA, Bondonno NP, Williams G, Teixeira-Pinto A, Craig JC, Wong G (2022). Exercise training for adults undergoing maintenance dialysis. Cochrane Database Syst Rev.

[53] Bennett PN, Bohm C, Harasemiw O, Brown L, Gabrys I, Jegatheesan D, Johnson DW, Lambert K, Lightfoot CJ, MacRae J (2022). Physical activity and exercise in peritoneal dialysis: International Society for Peritoneal Dialysis and the Global Renal Exercise Network practice recommendations. Perit Dial Int.

[54] Bennett PN, Bohm C, Yee-Moon Wang A, Kanjanabuch T, Figueiredo AE, Harasemiw O, Brown L, Gabrys I, Jegatheesan D, Lambert K (2023). An International Survey of Peritoneal Dialysis Exercise practices and perceptions. Kidney Int Rep.

[55] Hermsen S, Moons J, Kerkhof P, Wiekens C, De Groot M (2017). Determinants for sustained use of an activity Tracker: Observational Study. JMIR Mhealth Uhealth.

[56] Stauss M, Htay H, Kooman JP, Lindsay T, Woywodt A. Wearables in nephrology: fanciful gadgetry or Pret-a-Porter? Sens (Basel) 2023;23(3). 10.3390/s23031361 PMC9919296 36772401

[57] Mantoani LC, Rubio N, McKinstry B, MacNee W, Rabinovich RA (2016). Interventions to modify physical activity in patients with COPD: a systematic review. Eur Respir J.

[58] Franklin NC (2015). Technology to promote and increase physical activity in Heart Failure. Heart Fail Clin.

