# Supplementary material for: Objectively measured daily steps as an outcome in a clinical trial of chronic kidney disease: a systematic review
Source: BMC Nephrol. 2024 Jan 3;25:10. doi: 10.1186/s12882-023-03412-x (PMC10765814; doi:10.1186/s12882-023-03412-x)
Supplement: Supplementary file 1 — Supplementary Material 1: Table S1 The PRISMA 2020 Checklist. Table S2 Search detailed for databases. Table S3 Physical activity outcomes extracted from included studies. Table S4 Reasons for excluding studies from the final analysis after full text assessment. Figure S1 Results from three RCTs based on combined exercise training interventions. Figure S2 Results from three RCTs based on aerobic exercise training interventions. Figure S3 Results from three non-RCTs based on exercise training interventions. Figure S4 Results of three RCTs from a daily step goal-oriented interventions. Figure S5 Results of a single-arm trial from a daily step goal-oriented intervention. Figure S6 Results of an RCT from the mHealth combined exercise. Figure S7 Results from an RCT comparing different dialysis modalities [file 12882_2023_3412_MOESM1_ESM.docx]

**Objectively measured physical activity as an outcome in a clinical trial of full-spectrum chronic kidney disease**

**Supplementary Material**

**Catalog**

[Table S1 The PRISMA 2020 Checklist 2](#_Toc138194554)

[Table S2 Search detailed for databases. 6](#_Toc138194555)

[Table S3 Physical activity outcomes extracted from included studies. 9](#_Toc138194556)

[Table S4 Reasons for excluding studies from the final analysis after full text assessment. 12](#_Toc138194557)

[Figure S1 Results from three RCTs based on combined exercise training interventions. 18](#_Toc138194558)

[Figure S2 Results from three RCTs based on aerobic exercise training interventions. 19](#_Toc138194559)

[Figure S3 Results from three non-RCTs based on exercise training interventions. 20](#_Toc138194560)

[Figure S4 Results of three RCTs from a daily step goal-oriented interventions. 21](#_Toc138194561)

[Figure S5 Results of a single-arm trial from a daily step goal-oriented intervention. 22](#_Toc138194562)

[Figure S6 Results of an RCT from the mHealth combined exercise. 23](#_Toc138194563)

[Figure S7 Results from an RCT comparing different dialysis modalities. 24](#_Toc138194564)

[Reference 25](#_Toc138194565)

Table S1 The PRISMA 2020 Checklist

| **Section and Topic** | **Item #** | **Checklist item** | **Location where item is reported** |
| --- | --- | --- | --- |
| **TITLE** | | |  |
| Title | 1 | Identify the report as a systematic review. | Title page |
| **ABSTRACT** | | |  |
| Abstract | 2 | See the PRISMA 2020 for Abstracts checklist. | Page 2 |
| **INTRODUCTION** | | |  |
| Rationale | 3 | Describe the rationale for the review in the context of existing knowledge. | Page 3 |
| Objectives | 4 | Provide an explicit statement of the objective(s) or question(s) the review addresses. | Page 3 |
| **METHODS** | | |  |
| Eligibility criteria | 5 | Specify the inclusion and exclusion criteria for the review and how studies were grouped for the syntheses. | Page 5 |
| Information sources | 6 | Specify all databases, registers, websites, organisations, reference lists and other sources searched or consulted to identify studies. Specify the date when each source was last searched or consulted. | Page 5 and Table S2 |
| Search strategy | 7 | Present the full search strategies for all databases, registers and websites, including any filters and limits used. | Table S2 |
| Selection process | 8 | Specify the methods used to decide whether a study met the inclusion criteria of the review, including how many reviewers screened each record and each report retrieved, whether they worked independently, and if applicable, details of automation tools used in the process. | Page 5-6 |
| Data collection process | 9 | Specify the methods used to collect data from reports, including how many reviewers collected data from each report, whether they worked independently, any processes for obtaining or confirming data from study investigators, and if applicable, details of automation tools used in the process. | Page 5-6 |
| Data items | 10a | List and define all outcomes for which data were sought. Specify whether all results that were compatible with each outcome domain in each study were sought (e.g. for all measures, time points, analyses), and if not, the methods used to decide which results to collect. | Page 5-6 |
|  | 10b | List and define all other variables for which data were sought (e.g. participant and intervention characteristics, funding sources). Describe any assumptions made about any missing or unclear information. | Page 5-6 |
| Study risk of bias assessment | 11 | Specify the methods used to assess risk of bias in the included studies, including details of the tool(s) used, how many reviewers assessed each study and whether they worked independently, and if applicable, details of automation tools used in the process. | Page 6 |
| Effect measures | 12 | Specify for each outcome the effect measure(s) (e.g. risk ratio, mean difference) used in the synthesis or presentation of results. | Not applicable |
| Synthesis methods | 13a | Describe the processes used to decide which studies were eligible for each synthesis (e.g. tabulating the study intervention characteristics and comparing against the planned groups for each synthesis (item #5)). | Not applicable |
|  | 13b | Describe any methods required to prepare the data for presentation or synthesis, such as handling of missing summary statistics, or data conversions. |  |
|  | 13c | Describe any methods used to tabulate or visually display results of individual studies and syntheses. |  |
|  | 13d | Describe any methods used to synthesize results and provide a rationale for the choice(s). If meta-analysis was performed, describe the model(s), method(s) to identify the presence and extent of statistical heterogeneity, and software package(s) used. |  |
|  | 13e | Describe any methods used to explore possible causes of heterogeneity among study results (e.g. subgroup analysis, meta-regression). |  |
|  | 13f | Describe any sensitivity analyses conducted to assess robustness of the synthesized results. |  |
| Reporting bias assessment | 14 | Describe any methods used to assess risk of bias due to missing results in a synthesis (arising from reporting biases). | Not applicable |
| Certainty assessment | 15 | Describe any methods used to assess certainty (or confidence) in the body of evidence for an outcome. | Not applicable |
| **RESULTS** | | |  |
| Study selection | 16a | Describe the results of the search and selection process, from the number of records identified in the search to the number of studies included in the review, ideally using a flow diagram. | Page 5 |
|  | 16b | Cite studies that might appear to meet the inclusion criteria, but which were excluded, and explain why they were excluded. | Page 6 and Figure 1 |
| Study characteristics | 17 | Cite each included study and present its characteristics. | Table 1 |
| Risk of bias in studies | 18 | Present assessments of risk of bias for each included study. | Table 2 and Figure 2 |
| Results of individual studies | 19 | For all outcomes, present, for each study: (a) summary statistics for each group (where appropriate) and (b) an effect estimate and its precision (e.g. confidence/credible interval), ideally using structured tables or plots. | Table 1 |
| Results of syntheses | 20a | For each synthesis, briefly summarise the characteristics and risk of bias among contributing studies. | Not applicable |
|  | 20b | Present results of all statistical syntheses conducted. If meta-analysis was done, present for each the summary estimate and its precision (e.g. confidence/credible interval) and measures of statistical heterogeneity. If comparing groups, describe the direction of the effect. |  |
|  | 20c | Present results of all investigations of possible causes of heterogeneity among study results. |  |
|  | 20d | Present results of all sensitivity analyses conducted to assess the robustness of the synthesized results. |  |
| Reporting biases | 21 | Present assessments of risk of bias due to missing results (arising from reporting biases) for each synthesis assessed. | Not applicable |
| Certainty of evidence | 22 | Present assessments of certainty (or confidence) in the body of evidence for each outcome assessed. | Not applicable |
| **DISCUSSION** | | |  |
| Discussion | 23a | Provide a general interpretation of the results in the context of other evidence. | Page 11 |
|  | 23b | Discuss any limitations of the evidence included in the review. | Not applicable |
|  | 23c | Discuss any limitations of the review processes used. | Page 14 |
|  | 23d | Discuss implications of the results for practice, policy, and future research. | Page 13-14 |
| **OTHER INFORMATION** | | |  |
| Registration and protocol | 24a | Provide registration information for the review, including register name and registration number, or state that the review was not registered. | CRD42022385441 |
|  | 24b | Indicate where the review protocol can be accessed, or state that a protocol was not prepared. | Page 4 |
|  | 24c | Describe and explain any amendments to information provided at registration or in the protocol. | None |
| Support | 25 | Describe sources of financial or non-financial support for the review, and the role of the funders or sponsors in the review. | Page 15 |
| Competing interests | 26 | Declare any competing interests of review authors. | Page 15 |
| Availability of data, code and other materials | 27 | Report which of the following are publicly available and where they can be found: template data collection forms; data extracted from included studies; data used for all analyses; analytic code; any other materials used in the review. | Page 15 |

Table S2 Search detailed for databases.

| PubMed | | | | | | |  |
| --- | --- | --- | --- | --- | --- | --- | --- |
| # | Search detail | | | | | Results |  |
| 1 | "Renal Insufficiency, Chronic"[MeSH Terms] OR "Renal Insufficiency"[MeSH Terms] OR "Renal Replacement Therapy"[MeSH Terms] OR "Renal Insufficiency"[Title/Abstract] OR "Kidney Insufficiency"[Title/Abstract] OR "Kidney failure"[Title/Abstract] OR "Renal failure"[Title/Abstract] OR "Kidney disease"[Title/Abstract] OR "Renal disease"[Title/Abstract] OR "Predialysis"[Title/Abstract] OR "Pre-dialysis"[Title/Abstract] OR "End-Stage Kidney"[Title/Abstract] OR "End-Stage Renal"[Title/Abstract] OR "Endstage Kidney"[Title/Abstract] OR "Endstage Renal"[Title/Abstract] OR "Dialysis"[Title/Abstract] OR "Hemodialysis"[Title/Abstract] OR "Haemodialysis"[Title/Abstract] OR "Hemodiafiltration"[Title/Abstract] OR "Haemodiafiltration"[Title/Abstract] OR "Hemofiltration"[Title/Abstract] OR "Haemofiltration"[Title/Abstract] OR "Renal Transplantation"[Title/Abstract] OR "Kidney Grafting"[Title/Abstract] OR "Kidney Transplantation"[Title/Abstract] | | | | | 516974 |  |
| 2 | "Sedentary behavior"[MeSH Terms] OR "Metabolic Equivalent"[MeSH Terms] OR "Physical activity"[Title/Abstract] OR "Exercise volume"[Title/Abstract] OR "Step per day"[Title/Abstract] OR "Steps per day"[Title/Abstract] OR "Step count"[Title/Abstract] OR “Step/day" [Title/Abstract] OR "Steps/day"[Title/Abstract] OR "Step/d"[Title/Abstract] OR "Steps/d"[Title/Abstract] OR "Daily step"[Title/Abstract] OR "Daily steps" [Title/Abstract] OR "Sedentary Behavior"[Title/Abstract] OR "Sedentary Lifestyle" [Title/Abstract] OR "Physical Inactivity"[Title/Abstract] OR "Lack of Physical Activity"[Title/Abstract] OR "Sedentary Time"[Title/Abstract] OR "Metabolic Equivalent"[Title/Abstract] | | | | | 154673 |  |
| 3 | #1 AND #2 | | | | | 1968 |  |
| 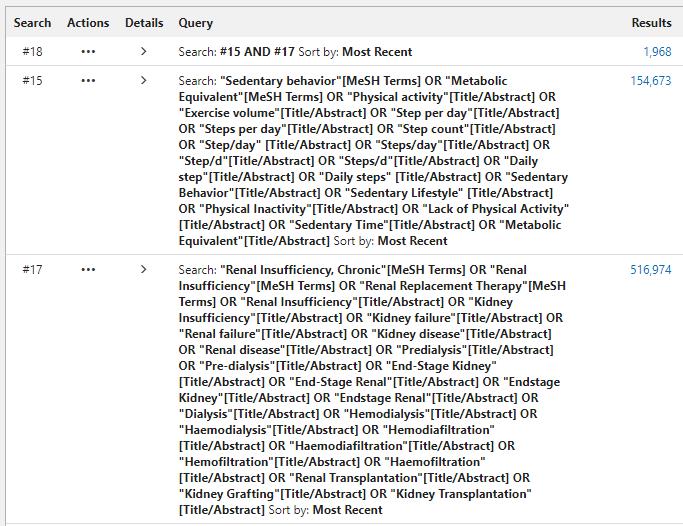 | | | | | | |  |
| Embase | | | | | | | |
| # | | Search detail | | | Results | | |
| 1 | | 'kidney disease'/exp OR "kidney disease":ti,ab,kw OR "renal disease":ti,ab,kw OR "kidney failure":ti,ab,kw OR "renal failure":ti,ab,kw OR "renal Insufficiency":ti,ab,kw OR "kidney Insufficiency":ti,ab,kw OR "end-stage kidney":ti,ab,kw OR "end-stage renal":ti,ab,kw OR "endstage kidney":ti,ab,kw OR "endstage renal":ti,ab,kw OR 'renal replacement therapy'/exp OR "dialysis":ti,ab,kw OR "hemodialysis":ti,ab,kw OR "haemodialysis":ti,ab,kw OR "hemofiltration":ti,ab,kw OR "haemofiltration":ti,ab,kw OR "hemodiafiltration":ti,ab,kw OR "haemodiafiltration":ti,ab,kw OR "renal transplantation":ti,ab,kw or "kidney grafting":ti,ab,kw OR "kidney transplantation":ti,ab,kw | | | 1419701 | | |
| 2 | | 'physical activity'/exp OR 'step count'/exp OR 'sedentary lifestyle'/exp OR 'metabolic equivalent'/exp OR "Physical activity":ti,ab,kw OR "Exercise volume":ti,ab,kw OR "Step per day":ti,ab,kw OR "Steps per day":ti,ab,kw OR "Step count":ti,ab,kw OR “Step/day":ti,ab,kw OR "Steps/day":ti,ab,kw OR "Step/d":ti,ab,kw OR "Steps/d":ti,ab,kw OR "Daily step":ti,ab,kw OR "Daily steps":ti,ab,kw OR "Sedentary Behavior":ti,ab,kw OR "Sedentary Lifestyle":ti,ab,kw OR "Physical Inactivity":ti,ab,kw OR "Lack of Physical Activity":ti,ab,kw OR "Sedentary Time":ti,ab,kw OR "Metabolic Equivalent":ti,ab,kw | | | 585109 | | |
| 3 | | #1 AND #2 | | | 11274 | | |
| 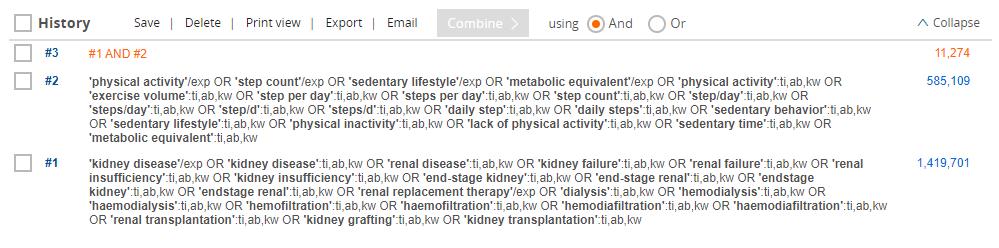 | | | | | | | |
| Web of Science | | | | | | | |
| # | | | Search detail | Results | | | |
| 1 | | | TS=("Renal Insufficiency" OR "Kidney Insufficiency" OR "Kidney failure" OR "Renal failure" OR "Kidney disease" OR "Renal disease" OR "Predialysis" OR "Pre-dialysis" OR "End-Stage Kidney" OR "End-Stage Renal" OR "Endstage Kidney" OR "Endstage Renal" OR "Dialysis" OR "Hemodialysis" OR "Haemodialysis" OR "Hemodiafiltration" OR "Haemodiafiltration" OR "Hemofiltration" OR "Haemofiltration" OR "Renal Transplantation" OR "Kidney Grafting" OR "Kidney Transplantation") | 446420 | | | |
| 2 | | | TS=("Physical activity" OR "Exercise volume" OR "Step per day" OR "Steps per day" OR "Step count" OR “Step/day" OR "Steps/day" OR "Step/d" OR "Steps/d" OR "Daily step" OR "Daily steps" OR "Sedentary Behavior" OR "Sedentary Lifestyle" OR "Physical Inactivity" OR "Lack of Physical Activity" OR "Sedentary Time" OR "Metabolic Equivalent") | 186437 | | | |
| 3 | | | #1 AND #2 | 2312 | | | |
| 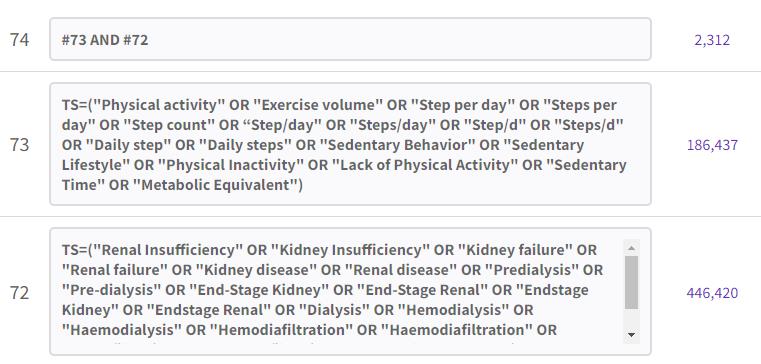 | | | | | | | |

Table S3 Physical activity outcomes extracted from included studies.

| **Author** |  | **Groups** | **Baseline** | **Midpoint** | **Endpoint** |
| --- | --- | --- | --- | --- | --- |
| Exercise training-based interventions | | | | | |
| Masajtis-Zagajewska A et al. (2019) [1] | Daily steps | CKD | 4091±2128 | 5924±2588 | 7291±2914 |
|  |  | KTR | 4400±2333 | 6847±2653 | 9093±3049 |
| Assawasaksakul N et al. (2021) [2] | Daily steps | I | 5574.6±4982.7 | - | 8250.1±3441.1 |
|  |  | C | 5183.4±3593.9 | - | 4671.7±3919.8 |
| Martins do Valle F et al. (2020) [3] ^†^ |  | I | 2520 (5734) | - | 1790 (6582) |
|  |  | C | 5975 (3713) | - | 4197 (2740) |
| Young HML et al. (2020) [4] | Daily steps (HD day) | I | 1373±1080 | - | 2444±1904 |
|  |  | C | 2252±4210 | - | 2464±4783 |
|  | Daily steps (non-HD day) | I | 2387±1696 | - | 2845±1696 |
|  |  | C | 3076±5790 | - | 2845±2117 |
| Watanabe K et al. (2021) [5] | Daily steps | I | 4820.5±2698.6 | 5710.7±2698.8 | 5316.8±2254.5 |
|  |  | C | 5817.9±3761.7 | 5032.5±3177.4 | 5011.3±3679.7 |
| Otobe Y et al. (2021) [6] ^†^ | Daily steps | I | 3540 (2061-6089) | - | 3757 (2324-6477) |
|  |  | C | 4171 (2688-6287) | - | 3575 (1923-5986) |
| Graham-Brow MPM et al. (2021) [7] | Daily steps | I | 2710±1945 | - | 3175±2610 |
|  |  | C | 3514±4099 | - | 3080±3445 |
| Hiraki K et al. (2017) [8] | Daily steps | I | 6725.3±3152.4 | - | 8281.4±3108.8 |
|  |  | C | 9113.0±3319.2 | - | 8828.8±2850.9 |
| Kontos P et al. (2020) [9] |  | Exercise-based | 5904±7199.41 | - | 3422.38±3052.73 |
|  |  | Film-based | 9227.22±6100.74 | - | 8571.44±7728.15 |
| Bulckaen M et al. (2011) [10] | Daily steps | Home-based | 2446±1642 | 3609±1978 | 4700±2366 |
|  |  | Supervised walking | 4353±2639 | 5446±2539 | 5130±2255 |
| Daily step goal-oriented interventions | | | | | |
| Sheshadri A et al. (2020) [11] | Daily steps | I | 3924±3422 | 5863±4019 | 4141±3737 |
|  |  | C | 3579±3680 | 3488±3292 | 4206±3736 |
| Nowicki M et al. (2010) [12] | Daily steps | 2 midweek dialysis sessions | 9337±5317 | - | 3766±1963 |
|  |  | Non-HD day | 11921±5909 | - | 4978±2495 |
| Malhotra R et al. (2023) [13] | Daily steps | I: Fitbit+feedback | 3704±1594 | 4830±2068  4617±2068 | 4624±2072 |
|  |  | C: Fitbit | 3808±1890 | 4302±2159  4083±2028 | 4089±2049 |
| O'Brien T et al. (2020) [14] ^*^ | Daily steps | I: SystemCHANGE™ + activity tracker | 4427±552 | 5139±533  5511±533  5674±533  5346±533  5436±533 | 4993±533 |
|  |  | C: Activity tracker | 4171±563 | 4865±543  4936±543  4710±543  4588±543  4566±543 | 4157±543 |
| Mobile health (mHealth) interventions | | | | | |
| Anand S et al. (2021) [15] | Daily steps | I: mHealth+exercise | 5943.5±4800.95 | 5212.7±4760.58 | 4997.5±4800.79 |
|  |  | C: Exercise | 5596.9±4904.21 | 5379.3±4710.4 | 4379.8±4858.21 |
| Different dialysis modalities | | | | | |
| Pecoits-Filho R et al. (2021) [16] | Daily steps | HDF | 5253±3062 | 5303±3442 | 4544±3131 |
|  |  | HD | 5045±3936 | 4249±2734 | 3986±3173 |
| Sit Less, Interact, Move More intervention | | | | | |
| Lyden K et al. (2021) [17] ^#^ | Daily steps | I: Sit Less, Interact, Move More intervention | 5142 (3766, 6741) | +537±570.24  +743±583.64  +1164±650.94  +1050±849.92  +1265±746.79 | +383±692.27 |
|  |  | C: standard care | 5363 (4115, 7631) | -163±580.26  294±880.12 | 103±423.45 |
| ***Note:*** Data are expressed as mean ± standard deviation, unless otherwise stated.  **Abbreviation:** I, intervention; C, control; HDF, hemodiafiltration; HD, hemodialysis; AE, aerobic exercise; RT, resistance training; CE, combined exercise  † Median (quartiles).  * Mean±standard error.  # Median (25th, 75th percentiles) and mean difference±standard deviation | | | | | |

Table S4 Reasons for excluding studies from the final analysis after full text assessment.

| **NO.** | **Reference** | **Reason for exclusion** |
| --- | --- | --- |
| 1 | Celano CM, Zambrano J, Harnedy L, et al: A novel health behaviour intervention to promote adherence in kidney failure. J Renal Care | Conference Abstracts |
| 2 | Junqué-Jiménez A, Esteve-Simó V, Andreu-Periz L, et al. A nurse-led home-based exercise program for patients with chronic kidney disease [published online ahead of print, 2022 Dec 1]. Worldviews Evid Based Nurs. 2022. | No physical activity outcome |
| 3 | Kim JC, Young Do J, Kang SH. Comparisons of physical activity and understanding of the importance of exercise according to dialysis modality in maintenance dialysis patients. Sci Rep. 2021;11(1):21487. | Non-clinical trials |
| 4 | Saipova D: Effect of 12 week trainig on physical activity on patient on a low protein diet. Nephrol Dial Transpl 2021, 36(SUPPL 1):i362. | Conference Abstracts |
| 5 | Ammar Y, Awad A: Effect of a supervised peri-dialytic exercise program on serum asymmetric dimethylarginine in maintenance hemodialysis patients. Nephrol Dial Transpl 2020, 35(SUPPL 3):iii1629. | Conference Abstracts |
| 6 | Bandi VK, Kolli H, Nimmagadda S, Komatla S: Effect of exercise on quality of life and functional capacity in patients with CKD. J Am Soc Nephrol 2020, 31:632. | Conference Abstracts |
| 7 | Sanad EA, El-Shinnawy HAEH, Hebah HA, et al. Effect of intra-dialytic physical exercise on depression in prevalent hemodialysis patients. Egyptian Journal of Neurology, Psychiatry and Neurosurgery 2022, 58(1). | Conference Abstracts |
| 8 | Hristea D, Deschamps T: Implementing an exercise program into the routine care of outpatient dialysis patients. Nephrol Dial Transpl 2016, 31:i478. | Conference Abstracts |
| 9 | Nowicki M, Zamojska S, Niewodniczy M, et al. Influence of intradialytic exercise training on habitual physical activity and nutritional parameters in chronic haemodialysis patients. Nephrol Dial Transpl 2006, 21:458-459. | Conference Abstracts |
| 10 | Ziolkowski S, Anand S, Bootwala AA, et al: A pilot randomized clinical trial to embed technology-enabled group-based exercise programming in the clinic: The exercise is medicine in chronic kidney disease trial. J Am Soc Nephrol 2020, 31:631-632. | Conference Abstracts |
| 11 | Kontos P, Alibhai SM, Miller KL, et al. A prospective 2-site parallel intervention trial of a research-based film to increase exercise amongst older hemodialysis patients. BMC Nephrol. 2017;18(1):37. | Protocol |
| 12 | Ortega-Pérez De Villar L, Martínez-Olmos FJ, Pérez-Domínguez FB, et al: Comparison of intradialytic versus home-based exercise programs on physical functioning, physical activity level, adherence, and health-related quality of life: pilot study. Sci Rep 2020, 10(1):8302. | Conference Abstracts |
| 13 | Myers J, Chan K, Chen Y, et al. Effect of a Home-Based Exercise Program on Indices of Physical Function and Quality of Life in Elderly Maintenance Hemodialysis Patients. Kidney Blood Press Res. 2021;46(2):196-206. | No physical activity outcome |
| 14 | Sheshadri A, Kittiskulnam P, Lai JC, et al. Effect of a pedometer-based intervention on body composition in ESRD. J Am Soc Nephrol 2019, 30:96. | Conference Abstracts |
| 15 | Reboredo MM, Valle FMD, Barros AAA, et al: Effects of intradialytic resistance training on physical activities in daily life and physical capacity in end-stage renal disease patients. Am J Resp Crit Care 2017, 195. | Conference Abstracts |
| 16 | Tobita I, Suzuki S, Kobayashi T, et al. A programme to encourage participation of haemodialysis patients in an exercise regimen. J Ren Care. 2009;35(1):48-53. | No physical activity outcome |
| 17 | Levendoğlu F, Altintepe L, Okudan N, et al. A twelve week exercise program improves the psychological status, quality of life and work capacity in hemodialysis patients. J Nephrol. 2004;17(6):826-832. | No physical activity outcome |
| 18 | Gomes TS, Aoike DT, Baria F, et al. Effect of Aerobic Exercise on Markers of Bone Metabolism of Overweight and Obese Patients With Chronic Kidney Disease. J Ren Nutr. 2017;27(5):364-371. | No physical activity outcome |
| 19 | Khalil E, Abdelaziz A, Zaki M, et al: Effect of aerobic programme during hemodialysis session on dialysis and cardiac hemodynamics in Egyptian hemodialysis patients on dialysis and cardiac hemodynamics in Egyptian hemodialysis patients. Nephrol Dial Transpl 2017, 32:i601. | Conference Abstracts |
| 20 | Cabrera-Pivaral CE, Bustamante-Rivera AP, Ramírez-Obeso RI, et al: Effect of an educational intervention to promote intradialysis aerobic excercises on the functional state of hemodialysis patients from Mexico. Revista de Nefrologia, Dialisis y Trasplante 2017, 37(4):207-214. | Non-English |
| 21 | Denguir S, Hellberg M, Rydell H, et al. Effects of baseline physical function and 12 months exercise training on survival in patients with non dialysis dependent chronic kidney disease. Nephrol Dial Transpl 2020, 35(SUPPL 3):i74. | Conference Abstracts |
| 22 | Maynard LG, de Menezes DL, Lião NS, et al. Effects of Exercise Training Combined with Virtual Reality in Functionality and Health-Related Quality of Life of Patients on Hemodialysis. Games Health J. 2019;8(5):339-348. | No physical activity outcome |
| 23 | Capitanini A, Cupisti A, Mochi N, et al. Effects of exercise training on exercise aerobic capacity and quality of life in hemodialysis patients. J Nephrol. 2008;21(5):738-743. | No physical activity outcome |
| 24 | Çetin Ç, Cebeci F. Effects of Web-Based Educational Intervention on Self-Management in Kidney Recipients. Exp Clin Transplant. 2018;16 Suppl 1(Suppl 1):117-118. | Review |
| 25 | Kubooka Y, Miuchi M, Shingaki H, et al: Efficacy of the intensive “activity” management coupled with “the patient diary” in the development of diabetic nephropathy. Diabetes Res Clin PR 2014, 106:S81. | Conference Abstracts |
| 26 | Stevenson JK, Campbell ZC, Webster AC, et al. eHealth interventions for people with chronic kidney disease. Cochrane Database Syst Rev. 2019;8(8):CD012379. | Review |
| 27 | Johansen KL, Sakkas GK, Doyle J, et al. Exercise counseling practices among nephrologists caring for patients on dialysis. Am J Kidney Dis. 2003;41(1):171-178. | Non-clinical trials |
| 28 | Greenwood SA, Koufaki P, Rush R, et al. British Renal Society Rehabilitation Network. Exercise counselling practices for patients with chronic kidney disease in the UK: a renal multidisciplinary team perspective. Nephron Clin Pract. 2014;128(1-2):67-72. | Non-clinical trials |
| 29 | Erica Y, Gustavo O, Hernando G, et al. Exercise during hemodialysis. A pilot study in a single center, Unidad Renal Sanitas. Bogota Colombia. Nephrol Dial Transpl 2017, 32:i601-i602. | Conference Abstracts |
| 30 | Kontos P, Grigorovich A, Colobong R, et al. Fit for Dialysis: a qualitative exploration of the impact of a research-based film for the promotion of exercise in hemodialysis. BMC Nephrol. 2018;19(1):195. | Non-clinical trials |
| 31 | Young HML, Jeurkar S, Churchward DR, et al. Implementing a theory-based intradialytic exercise programme in practice: a quality improvement project. Clin Kidney J. 2018;11(6):832-840. | No physical activity outcome |
| 32 | Atapour A, Vahdat S, Hosseini M, et al. Effect of Selenium on Triglyceride and Total Cholesterol, Weight Gain, and Physical Activity on Hemodialysis Patients: A Randomized Double-Blinded Controlled Trial [published correction appears in Int J Prev Med. 2022 Jun 24;13:83]. Int J Prev Med. 2022;13:63. | No physical activity outcome |
| 33 | Paglialonga F, Lopopolo A, Scarfia RV, et al: Intradialytic cycling in pediatric patients on chronic hemodialysis. Hemodial Int 2013, 17(1):163. | Conference Abstracts |
| 34 | Greenwood SA, Koufaki P, Macdonald JH, et al. Randomized Trial-PrEscription of intraDialytic exercise to improve quAlity of Life in Patients Receiving Hemodialysis. Kidney Int Rep. 2021;6(8):2159-2170. | Duplicate publication |
| 35 | Sheshadri A, Kittiskulnam P, Lai JC, et al. Effect of a pedometer-based walking intervention on body composition in patients with ESRD: a randomized controlled trial. BMC Nephrol. 2020;21(1):100. | Duplicate publication |
| 36 | Yamamoto S, Matsuzawa R, Kamitani T, et al. Efficacy of Exercise Therapy Initiated in the Early Phase After Kidney Transplantation: A Pilot Study. J Ren Nutr. 2020;30(6):518-525. | Non-clinical trials |
| 37 | Tao X, Zhang H, Lai L, et al. A 12-week personalised physical activity and dietary protein intervention for older adults undergoing peritoneal dialysis: A feasibility study. Geriatr Nurs. 2022;47:247-253. | Subjectively measured physical activity |
| 38 | Ortega-Pérez de Villar L, Martínez-Olmos FJ, Pérez-Domínguez FB, et al. Comparison of intradialytic versus home-based exercise programs on physical functioning, physical activity level, adherence, and health-related quality of life: pilot study. Sci Rep. 2020;10(1):8302. | Subjectively measured physical activity |
| 39 | Lendraitiene E, Lanevskaite E, Petrusevicienė D, et al. Effect of Different Physical Therapy Programs on Renal Transplant Recipients' Physical Activity, Grip Strength, and Psychoemotional Status and the Associations Between These Indices. Transplant Proc. 2018;50(10):3338-3345. | Subjectively measured physical activity |
| 40 | Muras-Szwedziak K, Masajtis-Zagajewska A, Pawłowicz E, et al. Effects of a Structured Physical Activity Program on Serum Adipokines and Markers of Inflammation and Volume Overload in Kidney Transplant Recipients. Ann Transplant. 2019;24:569-575. | Subjectively measured physical activity |
| 41 | Saitoh M, Ogawa M, Dos Santos MR, et al. Effects of Intradialytic Resistance Exercise on Protein Energy Wasting, Physical Performance and Physical Activity in Ambulatory Patients on Dialysis: A Single-Center Preliminary Study in a Japanese Dialysis Facility. Ther Apher Dial. 2016;20(6):632-638. | Subjectively measured physical activity |
| 42 | Segura-Ortí E, García-Testal A. Intradialytic virtual reality exercise: Increasing physical activity through technology. Semin Dial. 2019;32(4):331-335. | Subjectively measured physical activity |
| 43 | Beetham KS, Krishnasamy R, Stanton T, et al. Effect of a 3-Year Lifestyle Intervention in Patients with Chronic Kidney Disease: A Randomized Clinical Trial. J Am Soc Nephrol. 2022;33(2):431-441. | Subjectively measured physical activity |
| 44 | Chen JL, Godfrey S, Ng TT, et al. Effect of intra-dialytic, low-intensity strength training on functional capacity in adult haemodialysis patients: a randomized pilot trial. Nephrol Dial Transplant. 2010;25(6):1936-1943. | Subjectively measured physical activity |
| 45 | Perez-Dominguez B, Casaña-Granell J, Garcia-Maset R, et al. Effects of exercise programs on physical function and activity levels in patients undergoing hemodialysis: a randomized controlled trial. Eur J Phys Rehabil Med. 2021;57(6):994-1001. | Subjectively measured physical activity |
| 46 | Dong ZJ, Zhang HL, Yin LX. Effects of intradialytic resistance exercise on systemic inflammation in maintenance hemodialysis patients with sarcopenia: a randomized controlled trial. Int Urol Nephrol. 2019;51(8):1415-1424. | Subjectively measured physical activity |
| 47 | Ammar YA, Awad A. Effect of a Supervised Peridialytic Exercise Program on Serum Asymmetric Dimethylarginine in Maintenance Hemodialysis Patients. Int J Nephrol. 2020;2020:8878306. | Subjectively measured physical activity |
| 48 | Koh KP, Fassett RG, Sharman JE, et al. Effect of intradialytic versus home-based aerobic exercise training on physical function and vascular parameters in hemodialysis patients: a randomized pilot study. Am J Kidney Dis. 2010;55(1):88-99. | Subjectively measured physical activity |
| 49 | Painter PL, Hector L, Ray K, et al. A randomized trial of exercise training after renal transplantation. Transplantation. 2002;74(1):42-48. | Subjectively measured physical activity |
| 50 | Alahnoori F, Toulabi T, Kordestani-Moghadam P, et al. The Effect of Sumac Fruit on Serum Lipids and Body Mass Index in Hemodialysis Patients. Evid Based Complement Alternat Med. 2022;2022:1687740. | Subjectively measured physical activity |
| 51 | Anand S, Ziolkowski SL, Bootwala A, et al. Group-Based Exercise in CKD Stage 3b to 4: A Randomized Clinical Trial. Kidney Med. 2021;3(6):951-961.e1. | Subjectively measured physical activity |
| 52 | Greenwood SA, Koufaki P, Macdonald JH, et al. Exercise programme to improve quality of life for patients with end-stage kidney disease receiving haemodialysis: the PEDAL RCT. Health Technol Assess. 2021;25(40):1-52. | Subjectively measured physical activity |
| 53 | Cho JH, Lee JY, Lee S, et al. Effect of intradialytic exercise on daily physical activity and sleep quality in maintenance hemodialysis patients. Int Urol Nephrol. 2018;50(4):745-754. | Non-daily steps |
| 54 | Bohm C, Stewart K, Onyskie-Marcus J, et al. Effects of intradialytic cycling compared with pedometry on physical function in chronic outpatient hemodialysis: a prospective randomized trial. Nephrol Dial Transplant. 2014;29(10):1947-1955. | Non-daily steps |
| 55 | Kirkman DL, Ramick MG, Muth BJ, et al. A randomized trial of aerobic exercise in chronic kidney disease: Evidence for blunted cardiopulmonary adaptations. Ann Phys Rehabil Med. 2021;64(6):101469. | Non-daily steps |
| 56 | Gibson CA, Gupta A, Greene JL, et al. Feasibility and acceptability of a televideo physical activity and nutrition program for recent kidney transplant recipients. Pilot Feasibility Stud. 2020;6:126. | Non-daily steps |
| 57 | Pike MM, Alsouqi A, Headley SAE, et al. Supervised Exercise Intervention and Overall Activity in CKD. Kidney Int Rep. 2020;5(8):1261-1270. | Non-daily steps |
| 58 | Turoń-Skrzypińska A, Dutkiewicz G, Marchelek-Myśliwiec M, et al. Physical Activity versus Sclerostin and Interleukin 6 Concentration in Patients Receiving Renal Replacement Therapy by Hemodialysis. Risk Manag Healthc Policy. 2020;13:1467-1475. | Non-clinical trials |


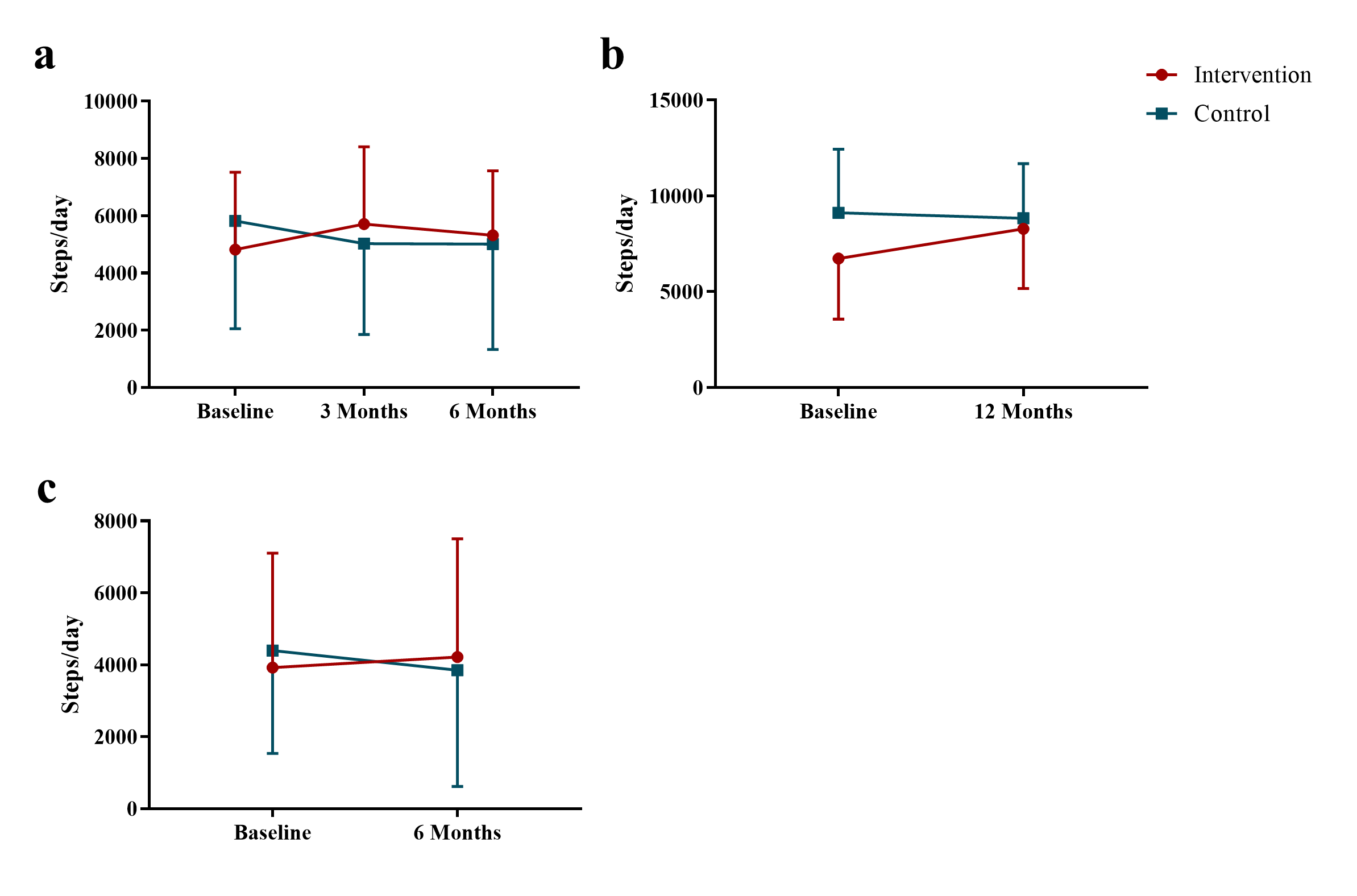


Figure S1 Results from three RCTs based on combined exercise training interventions.

a) Watanabe K et al.; b) Hiraki K et al.; c) Otobe Y et al.


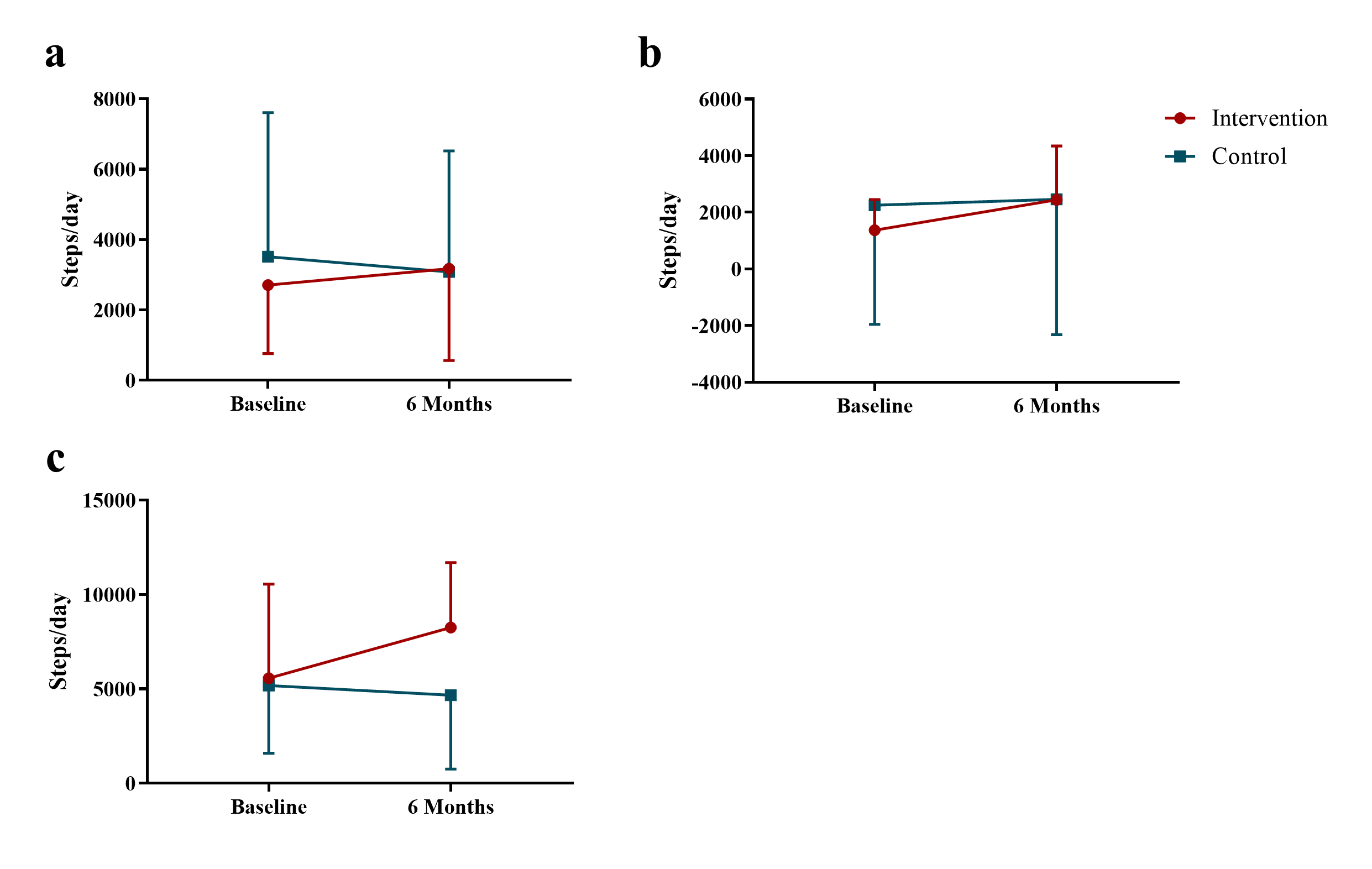


Figure S2 Results from three RCTs based on aerobic exercise training interventions.

a) Graham-Brown MPM et al.; b) Young HML et al.; c) Assawasaksakul N et al.


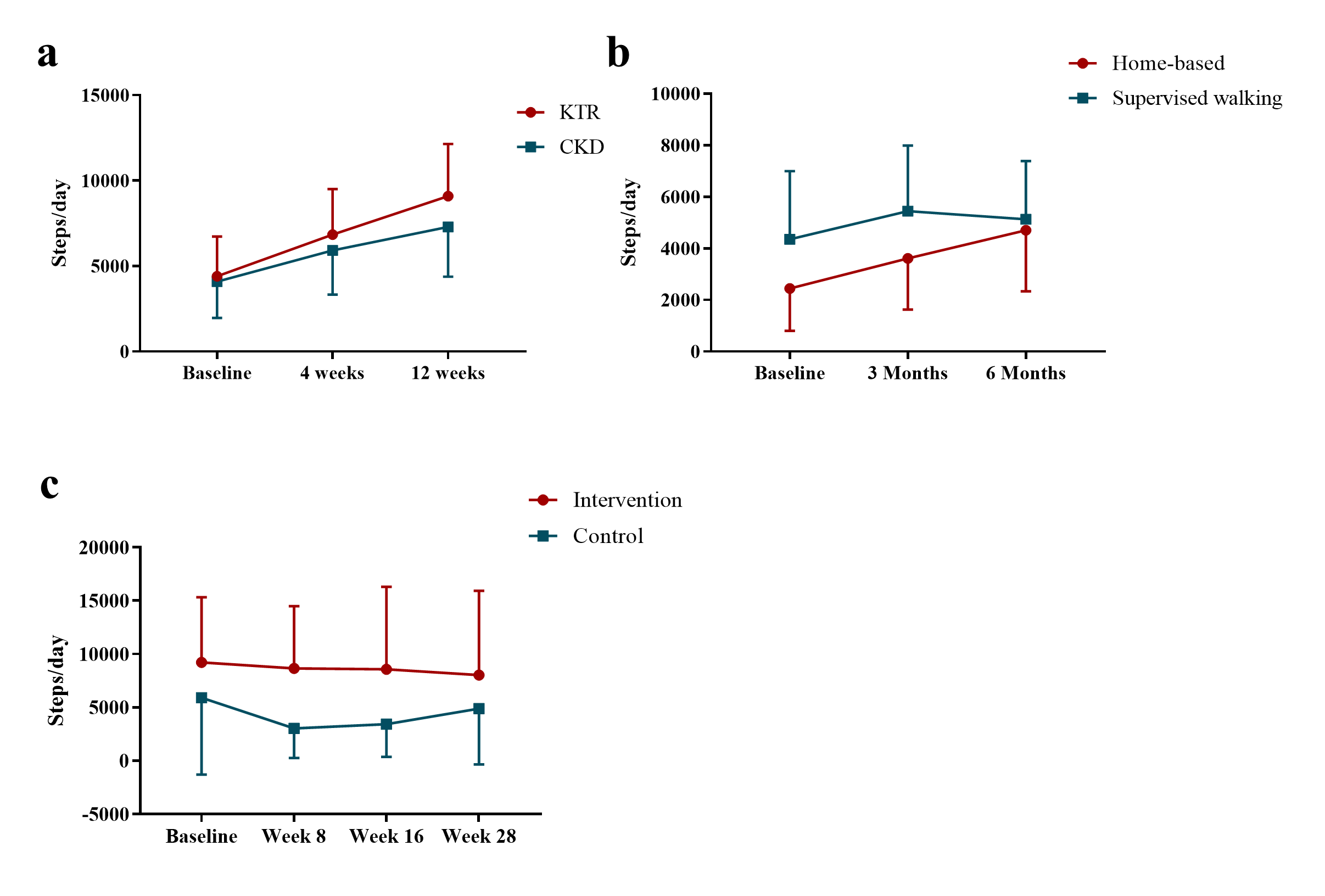


Figure S3 Results from three non-RCTs based on exercise training interventions.

a) Masajtis-Zagajewska A et al.; b) Bulckaen M et al.; c) Kontos P et al.


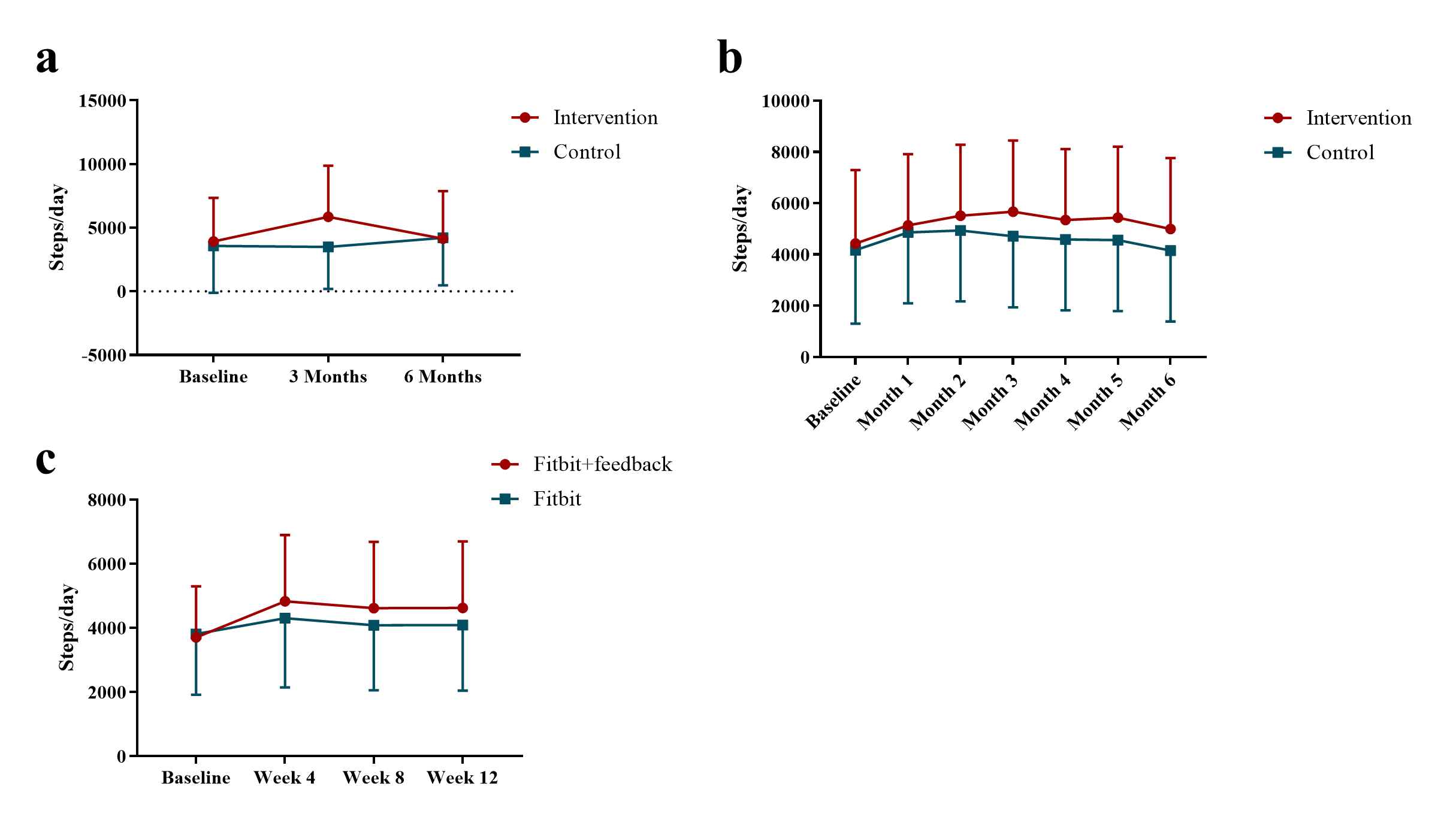


Figure S4 Results of three RCTs from a daily step goal-oriented interventions.

a) Sheshadri A et al.; b) O'Brien T et al.; c) Malhotra R et al.


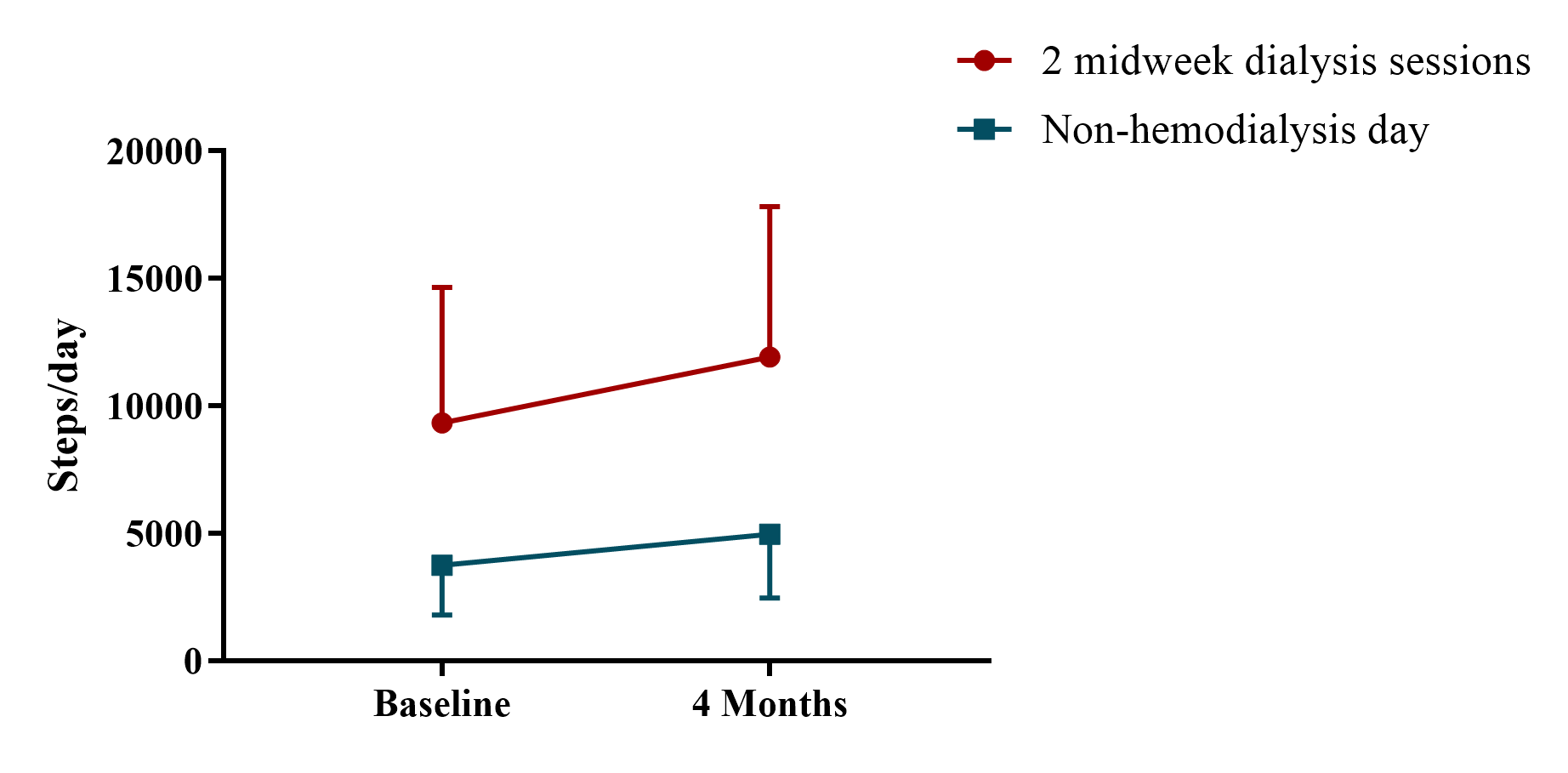


Figure S5 Results of a single-arm trial from a daily step goal-oriented intervention.


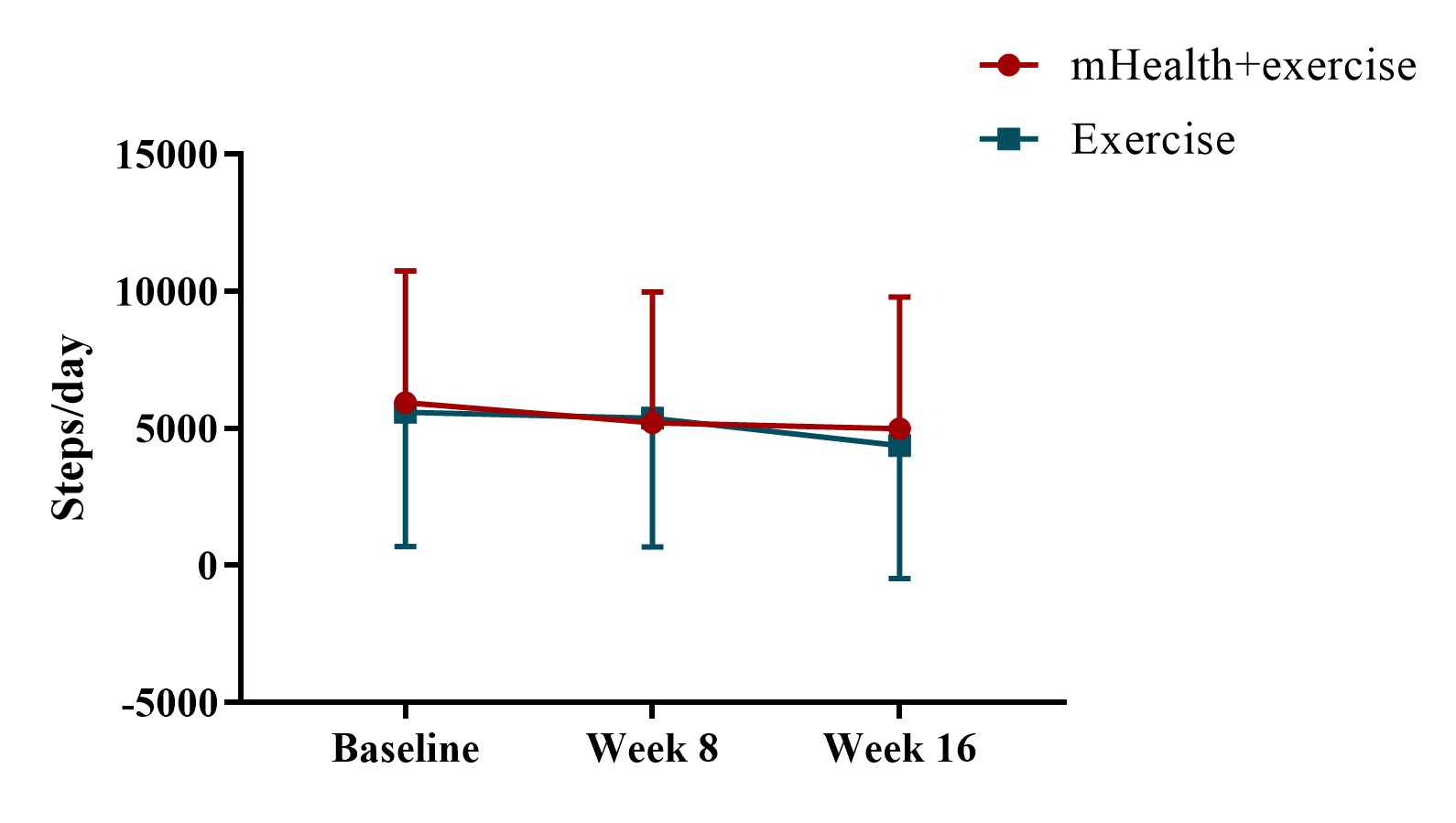


Figure S6 Results of an RCT from the mHealth combined exercise.


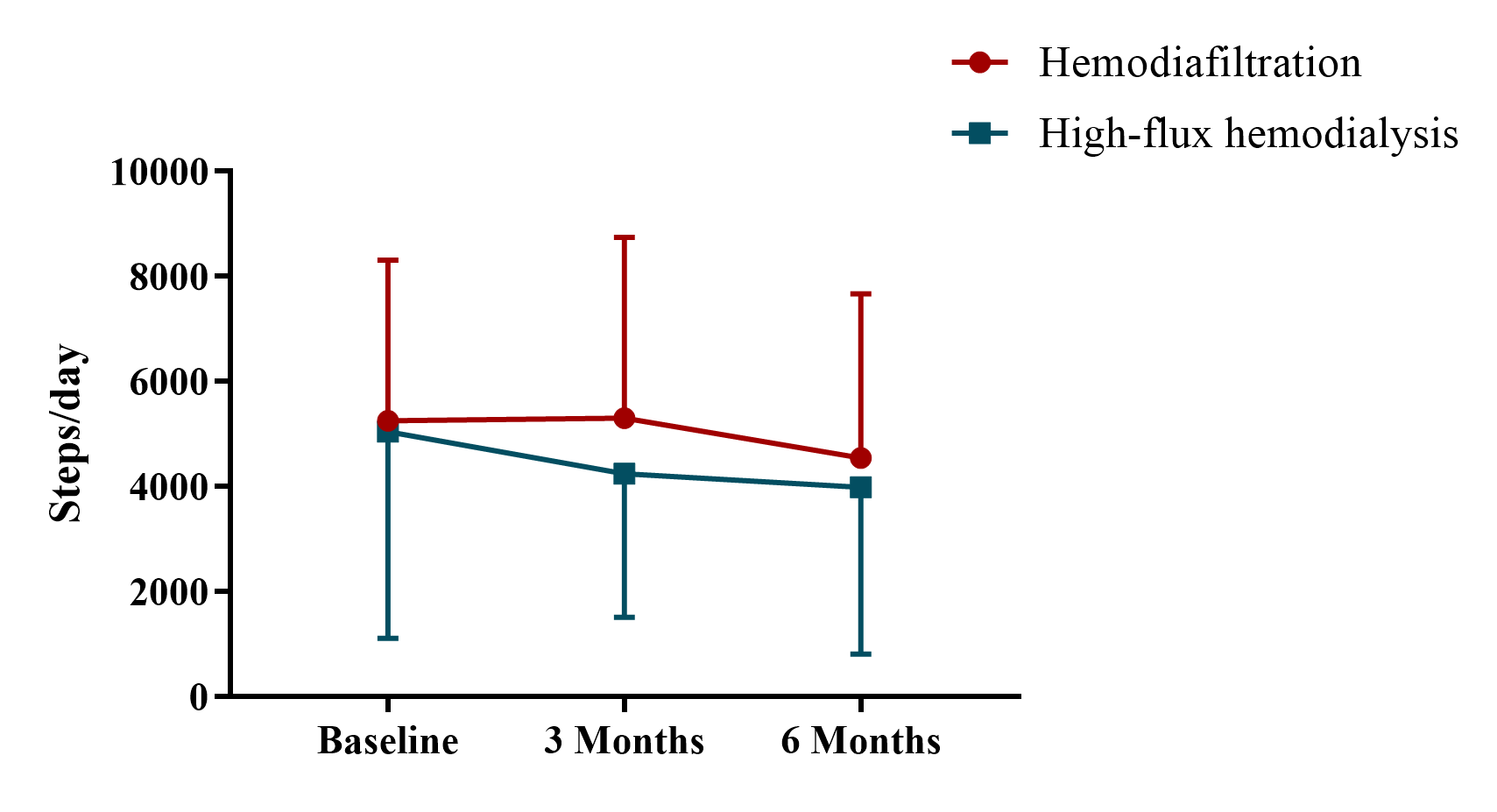
Figure S7 Results from an RCT comparing different dialysis modalities.

Reference

1. Masajtis-Zagajewska A, Muras K, Nowicki M: **Effects of a Structured Physical Activity Program on Habitual Physical Activity and Body Composition in Patients With Chronic Kidney Disease and in Kidney Transplant Recipients**. *Exp Clin Transplant* 2019, **17**(2):155-164.

2. Assawasaksakul N, Sirichana W, Joosri W, Kulaputana O, Eksakulkla S, Ketanun C, Kittiskulnam P, Chantadisai M, Takkavatakarn K, Susantitaphong P *et al*: **Effects of intradialytic cycling exercise on daily physical activity, physical fitness, body composition, and clinical parameters in high-volume online hemodiafiltration patients: a pilot randomized-controlled trial**. *Int Urol Nephrol* 2021, **53**(2):359-371.

3. Martins do Valle F, Valle Pinheiro B, Almeida Barros AA, Ferreira Mendonça W, de Oliveira AC, de Oliveira Werneck G, de Paula RB, Moura Reboredo M: **Effects of intradialytic resistance training on physical activity in daily life, muscle strength, physical capacity and quality of life in hemodialysis patients: a randomized clinical trial**. *Disabil Rehabil* 2020, **42**(25):3638-3644.

4. Young HML, March DS, Highton PJ, Graham-Brown MPM, Churchward DC, Grantham C, Goodliffe S, Jones W, Cheung MM, Greenwood SA *et al*: **Exercise for people living with frailty and receiving haemodialysis: a mixed-methods randomised controlled feasibility study**. *BMJ Open* 2020, **10**(11):e041227.

5. Watanabe K, Kamijo Y, Yanagi M, Ishibashi Y, Harada T, Kohzuki M: **Home-based exercise and bone mineral density in peritoneal dialysis patients: a randomized pilot study**. *BMC Nephrol* 2021, **22**(1):98.

6. Otobe Y, Yamada M, Hiraki K, Onari S, Taki Y, Sumi H, Hachisuka R, Han W, Takahashi M, Suzuki M *et al*: **Physical Exercise Improves Cognitive Function in Older Adults with Stage 3-4 Chronic Kidney Disease: A Randomized Controlled Trial**. *Am J Nephrol* 2021, **52**(12):929-939.

7. Graham-Brown MPM, March DS, Young R, Highton PJ, Young HML, Churchward DR, Dungey M, Stensel DJ, Bishop NC, Brunskill NJ *et al*: **A randomized controlled trial to investigate the effects of intra-dialytic cycling on left ventricular mass**. *Kidney Int* 2021, **99**(6):1478-1486.

8. Hiraki K, Shibagaki Y, Izawa KP, Hotta C, Wakamiya A, Sakurada T, Yasuda T, Kimura K: **Effects of home-based exercise on pre-dialysis chronic kidney disease patients: a randomized pilot and feasibility trial**. *BMC Nephrol* 2017, **18**(1):198.

9. Kontos P, Colobong R, Grigorovich A, Palma Lazgare LI, Binns M, Alibhai S, Parsons T, Nesrallah G, Jassal SV, Thomas A *et al*: **Fit for Dialysis: a prospective 2-site parallel intervention trial of a filmed research-based drama to increase exercise amongst older hemodialysis patients**. *Int Urol Nephrol* 2021, **53**(6):1223-1230.

10. Bulckaen M, Capitanini A, Lange S, Caciula A, Giuntoli F, Cupisti A: **Implementation of exercise training programs in a hemodialysis unit: effects on physical performance**. *J Nephrol* 2011, **24**(6):790-797.

11. Sheshadri A, Kittiskulnam P, Lazar AA, Johansen KL: **A Walking Intervention to Increase Weekly Steps in Dialysis Patients: A Pilot Randomized Controlled Trial**. *Am J Kidney Dis* 2020, **75**(4):488-496.

12. Nowicki M, Murlikiewicz K, Jagodzińska M: **Pedometers as a means to increase spontaneous physical activity in chronic hemodialysis patients**. *J Nephrol* 2010, **23**(3):297-305.

13. Malhotra R, Rahimi S, Agarwal U, Katz R, Kumar U, Garimella PS, Gupta V, Chopra T, Kotanko P, Ikizler TA *et al*: **The Impact of a Wearable Activity Tracker and Structured Feedback Program on Physical Activity in Hemodialysis Patients: The Step4Life Pilot Randomized Controlled Trial**. *Am J Kidney Dis* 2023.

14. O'Brien T, Russell CL, Tan A, Mion L, Rose K, Focht B, Daloul R, Hathaway D: **A Pilot Randomized Controlled Trial Using SystemCHANGE™ Approach to Increase Physical Activity in Older Kidney Transplant Recipients**. *Prog Transplant* 2020, **30**(4):306-314.

15. Anand S, Ziolkowski SL, Bootwala A, Li J, Pham N, Cobb J, Lobelo F: **Group-Based Exercise in CKD Stage 3b to 4: A Randomized Clinical Trial**. *Kidney Med* 2021, **3**(6):951-961 e951.

16. Pecoits-Filho R, Larkin J, Poli-de-Figueiredo CE, Cuvello-Neto AL, Barra ABL, Gonçalves PB, Sheth S, Guedes M, Han M, Calice-Silva V *et al*: **Effect of hemodiafiltration on measured physical activity: primary results of the HDFIT randomized controlled trial**. *Nephrol Dial Transplant* 2021, **36**(6):1057-1070.

17. Lyden K, Boucher R, Wei G, Zhou N, Christensen J, Chertow GM, Greene T, Beddhu S: **Targeting Sedentary Behavior in CKD: A Pilot and Feasibility Randomized Controlled Trial**. *Clin J Am Soc Nephrol* 2021, **16**(5):717-726.
